# External Stimuli‐Activable Single‐Atom Nanozymes for Bioapplications

**DOI:** 10.1002/smll.73447

**Published:** 2026-04-20

**Authors:** Hyun Ko, Jungsue Choi, Hyeonjeong Noh, Sohyeon Seo, Heedong Kwon, Seungjune Lee, Hyoyoung Lee

**Affiliations:** ^1^ Department of Biophysics Sungkyunkwan University (SKKU) Suwon Republic of Korea; ^2^ Institute of Quantum Biophysics Sungkyunkwan University (SKKU) Suwon Republic of Korea; ^3^ Department of Chemistry Sungkyunkwan University (SKKU) Suwon Republic of Korea; ^4^ National Institute of Climate and Environment (NICE) Sungkyunkwan University (SKKU) Suwon Republic of Korea

**Keywords:** bioapplication, external stimulation, nanozymes, single‐atom

## Abstract

Single‐atom nanozymes (SANs) emerge as promising alternatives to natural enzymes by precisely dispersing and controlling metal active sites at the atomic level, thereby maximizing catalytic site exposure and mimicking enzyme‐like structures. Recently, strategies that integrate SANs with external stimuli to enhance catalytic efficiency or selectively trigger activation are attracting increasing attention in various biomedical fields. However, the underlying mechanisms of the performance enhancement of SANs through external stimuli remain unexplored. Here, we discuss the engineering principles and strategies to improve the performance of SANs, with a particular emphasis on stimuli‐activable systems. Specifically, we review how external stimuli (such as light, ultrasound, and magnetic fields) can modulate the electronic structure of active sites and the physicochemical properties of supporting materials, thereby enhancing charge transfer, substrate activation, and reactive oxygen species generation. Furthermore, we provide an overview of the latest applications of stimuli‐activable SANs in biosensing, cancer therapy, tissue regeneration, and theranostics platforms. Lastly, we provide the prospects, challenges, and opportunities for external‐stimuli SANs.

## Graphical abstract

Stimuli‐activable single‐atom nanozymes (SANs) are reviewed from materials design to biomedical applications. We explain electronic‐structure tuning principles and synthesis strategies that maximize the responsiveness of metal active sites and supports to light, ultrasound, and magnetic fields. We conclude with case studies in bio‐sensing, therapy, Tissue regeneration, and theranostics that highlight opportunities and remaining challenges.

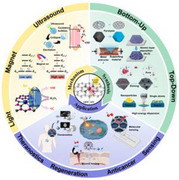

## 1 Introduction

Natural enzymes, with their outstanding substrate specificity and rapid catalytic rates, have played essential roles in industrial catalysis and biomedical applications. However, their practical use is limited by intrinsic constraints such as structural instability under harsh conditions, challenges in reuse, reduced long‐term storage stability, laborious formulation, and high production cost [1, 2]. To overcome these limitations, efforts to artificially mimic enzymes have continued since the 1970s [3]. In the 2000s, Scrimin et al. coined the term nanozyme to describe nanomaterials that exhibit enzyme‐like activities, stimulating major advances in artificial enzyme research [4, 5]. Since then, a wide range of nanomaterials, including have been reported to display oxidase (OXD), peroxidase (POD), catalase (CAT), superoxide dismutase (SOD), glucose oxidase (GOx), and glutathione peroxidase (GPx) like activities [6, 7, 8, 9]. More recently, enabled by nanotechnology that allows atom‐level synthesis and precise characterization, single‐atom catalyst‐based nanozymes with enzyme‐like activities (single‐atom nanozymes, SANs) have emerged. SANs individually anchored metal atoms on supporting materials to mimic the active sites of natural enzymes at the atomic scale, offering a promising route to high activity and selectivity [10, 11, 12]. However, SANs still require improvement in their catalytic efficiency, inferior to that of natural enzymes. The difficulty of precisely regulating reaction pathways and reactive oxygen species (ROS) production compromises controllability and raises concerns about cytotoxicity. These limitations highlight the need for new strategies that enable precise control while achieving high activity and selectivity. A recent study of externally stimuli‐activable SANs is providing rational strategies to enhance control and efficiency [13, 14].

Utilizing external stimuli enables accelerating and remotely controlling the catalytic activity of SANs, such as light, ultrasound, electric and magnetic fields, temperature, X‐rays, and microwaves [15, 16, 17, 18, 19]. Although various external stimuli have been explored in nanozyme research, studies on SANs have primarily relied on light, ultrasound, and magnetic fields. These three stimuli consistently provide practical applicability and clear mechanistic responses. Therefore, this review focuses on them. Stimuli‐activable approaches enable wireless or non‐invasive activation, offering superior selectivity, minimal invasiveness, and negligible side effects compared with conventional methods [13, 20, 21, 22, 23]. The rational design and synthesis of SANs capable of synergistically responding to external fields hold significant promise for overcoming existing limitations. Moreover, notable progress has been made by engineering the electronic, chemical, and optical properties of both active sites and supporting materials to maximize catalytic synergy [24]. Nonetheless, the systematic integration of SANs with external stimuli remains underexplored, despite their great potential to unlock new levels of catalytic performance. Several reviews have covered SANs, and numerous reviews have also discussed nanozymes that respond to external stimuli. However, no review has specifically addressed stimuli‐activable SANs. This review fills this gap by bringing together SANs activated by light, ultrasound, and magnetic fields and by outlining the fundamental principles underlying their stimulus‐driven activation.

In this paper, we review recent advances in external‐stimulus‐activable SANs, focusing on design principles and activation mechanisms for biomedical applications, based on theoretical and experimental results. First, design principles were discussed for tailoring supporting materials to amplify these effects, and then examining how tuning the metal species and atomic configuration was investigated to enhance synergy with different stimulus energies. Next, an in‐depth understanding of the activation mechanisms provides the guide for the catalytic exploration and ideal synthesis of stimuli‐activable SANs [25]. Then, representative achievements were highlighted in the application of stimuli‐activable SANs for tumour therapy, antibacterial treatments, and anti‐inflammatory interventions. Finally, remaining challenges and future directions were considered for advancing stimuli‐activable SANs‐based biomedical nanotechnologies toward practical clinical translation.

## 2 Functional Configuration of Stimuli‐Activable SANs

SANs are defined by a metal active site precisely anchored within highly engineered coordination environments, which play a decisive role in shaping their unique electronic structures and catalytic reactivity [26, 27]. Strong coordination bonds between the metal atoms and their supporting materials effectively suppress aggregation and enable tuning of electronic properties such as charge density, oxidation, spin state, and metal d band state, thus improving catalytic activity and selectivity [28, 29]. In particular, charge transfer and orbital hybridization between the metal active site d band state and the supporting materials modify the electronic structure, which in turn affects its adsorption binding energies and reaction pathways [30]. Accordingly, atomic‐level modulations of the coordination environment are critical not only for designing highly active SANs but also for elucidating their reaction mechanisms. Typically, the coordination environment of SANs comprises a first coordination sphere directly bonded to the metal center, a second coordination sphere arising from interactions with adjacent support atoms, and a broader hierarchical support framework at the nanoscale. Among these, the first coordination shell strongly influences the electronic structure depending on the type and coordination number of adjacent atoms (e.g., N, O, S) [30, 31, 32]. For example, diverse configurations such as M‐N_x_, mixed M‐N/O/S sites, and M‐X can induce distinct oxidation states, charge, and spin states on the metal active site, so adsorbate binding and pathway selectivity [33, 34, 35, 36]. Under suitable substrates and conditions, these effects can be leveraged to modulate ROS‐generation routes and enzyme‐like activities. The second coordination sphere and the physicochemical properties of the support offer an additional layer of control [37, 38]. Tailoring the local microenvironment via heteroatom doping, vacancy engineering, or polar functional groups can further regulate charge transfer, spin polarization, and the stabilization of key intermediates [39, 40]. Taken together, these engineered microenvironments determine activity and selectivity and provide a foundation for extending SANs control to externally driven regulation.

**FIGURE 1:**
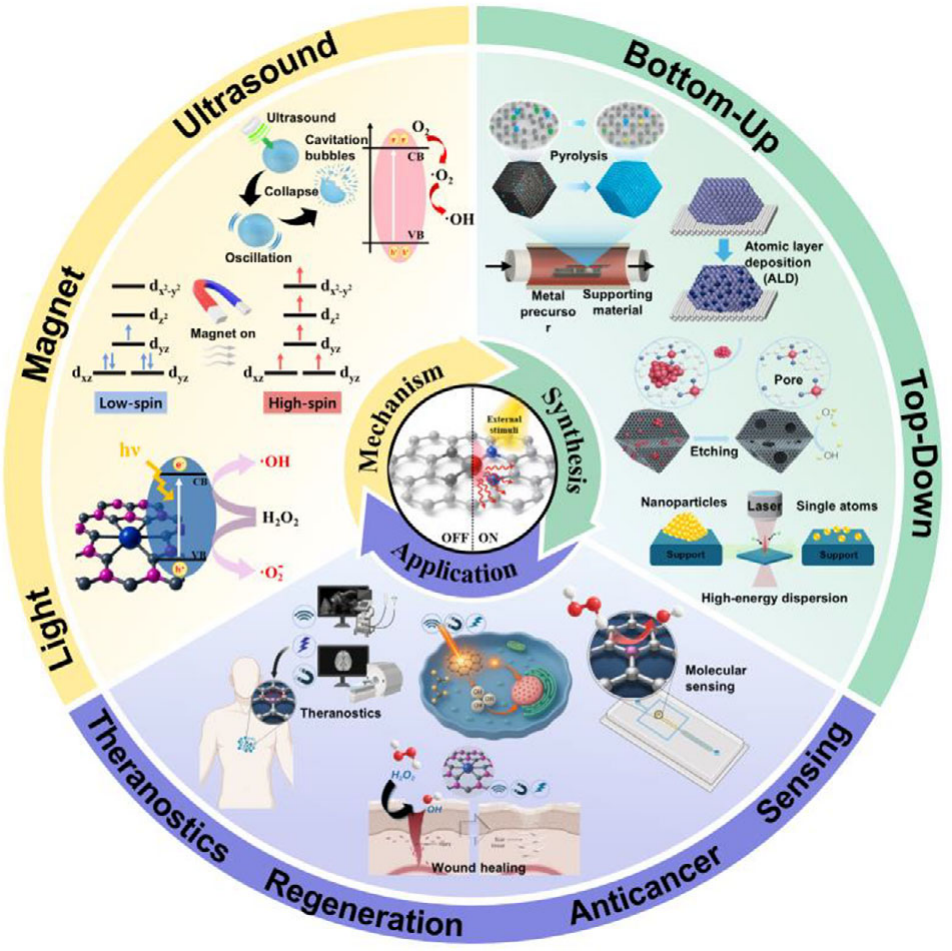
Schematic illustration of external stimuli‐activable single‐atom nanozymes (SANs) for mechanism, synthesis, and bioapplication.

The ability to tailor these coordination environments is important when designing SANs that are responsive to external physical stimuli. These engineered environments enable tunable control over catalytic behavior under external triggers by modulating spin polarization and ordering, charge separation, and radical intermediate formation. Thus, tuning the coordination environment to manipulate the electronic structure of single‐atom centers is a key strategy for developing stimuli‐activable SANs systems. To achieve strong responses to external stimuli, a reaction‐specific metal center must be robustly anchored on stimulus‐responsive supporting materials. Maximizing this responsiveness also requires careful consideration of the supporting material's physicochemical properties. Responsive supporting material whose electronic structures can be modulated by external inputs through charge transfer, thermal conduction, or acoustic effects can simultaneously enhance the operational efficiency and stimulus activation of SANs [41, 42, 43, 44]. For example, N‐doped carbon or TiO_2_ with oxygen vacancies can strengthen electron‐hole separation under light irradiation, amplifying catalytic reactions at the metal active site [45, 46, 47, 48]. Magnetically responsive supporting materials can efficiently transduce applied magnetic fields into magnetothermal and magnetomechanical stimuli (e.g., Fe_3_O_4_) [49, 50]. A magnetic field can induce spin polarization at a single‐atom metal active site, such as Fe, Co, Ru, and Gd [14, 51]. This change in spin ordering modulates the spin‐selective reaction pathway and the reactivity of the active site. Moreover, 2D supporting materials such as reduced graphene oxide, MXenes, or transition metal dichalcogenides (e.g., MoS_2_) can harness ultrasound cavitation to induce mechanical impacts or tailor electron transfer pathways, facilitating or regulating ROS generation [52, 53, 54, 55]. Overall, effective control of the catalytic activity of SANs responsive to external stimuli requires an integrated design that considers both the atomic coordination of the metal center and the physicochemical properties of the support. This integration renders stimulus sensitivity, catalytic activity, and ROS output tunable. It enables applications across diverse biomedical areas, including biosensing, antimicrobial therapy, tumor treatment, wound healing, and imaging‐guided diagnostics (Figure 1) [56, 57, 58, 59].

## 3 Stimuli‐Activable Mechanisms of SANs

### 3.1 Light‐Activable SANs

#### 3.1.1 Supporting Material Engineering for Light‐Harvesting

**FIGURE 2:**
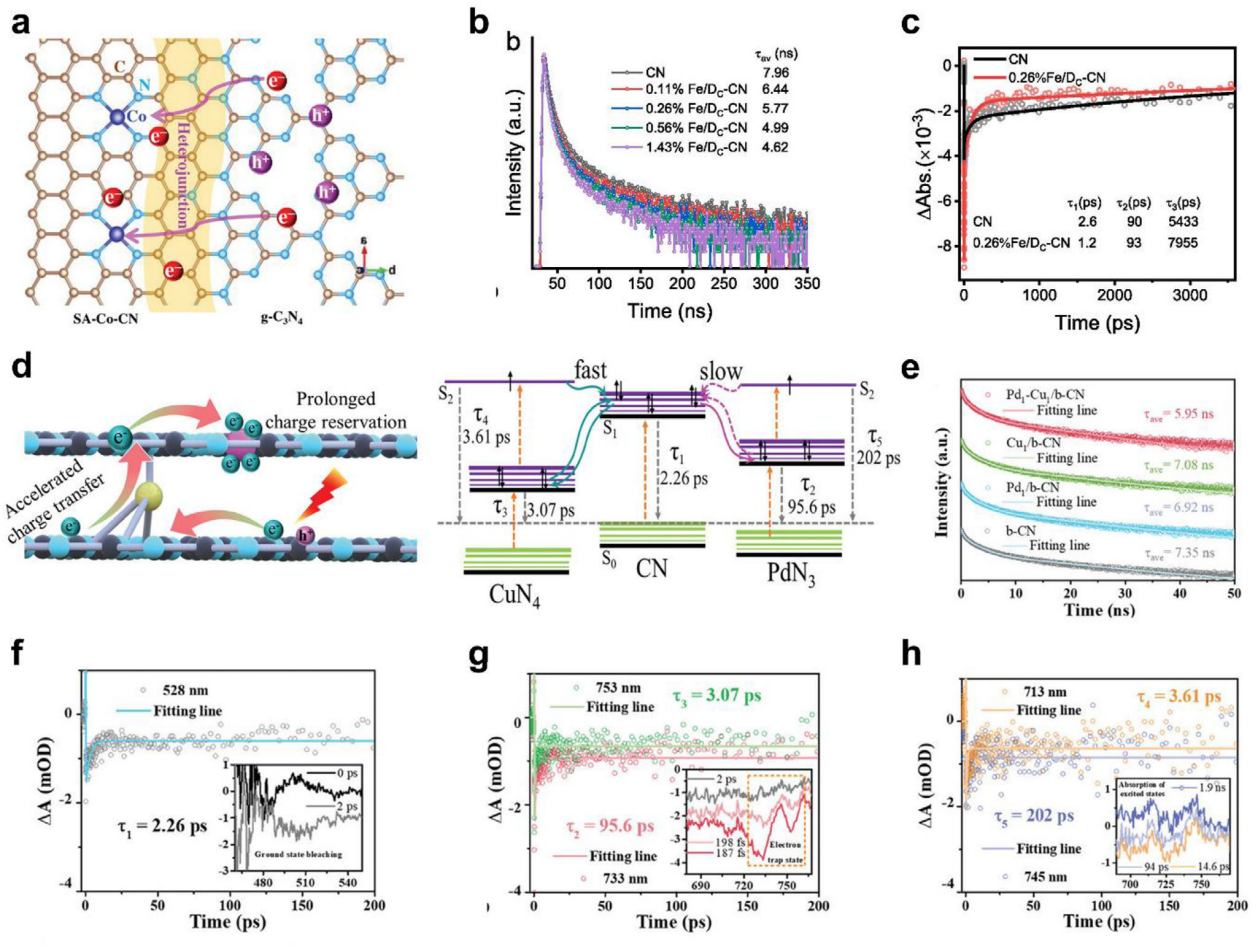
Light‐activable SANs. (a) Schematic illustration of the charge transfer between the heterojunction of SA‐Co‐CN and g‐C_3_N_4_. Reproduced with permission [63]. Copyright 2023, Wiley‐VCH GmbH. (b) Time‐resolved photothermal deflection spectra of CN and Fe/DC‐CN. (c) Transient absorption (TA) spectra of CN and 0.26% Fe/DC‐CN, the corresponding TA kinetics at 573 nm. Reproduced with permission [65]. Copyright 2025, Elsevier B.V. (d) Schematic illustration of dynamic charge transfer on Pd_1_‐Cu_1_/b‐CN during photocatalysis and cascade functions over interplanar and in‐plane dual single‐atoms in layer‐stacked bulk‐like g‐C_3_N_4_. (e) Time‐resolved transient Photoluminescence (TRPL) decay of CN, Pd_1_/b‐CN, Cu_1_/b‐CN, and Pd_1_‐Cu_1_/b‐CN. TA kinetic traces probed at f) 528, g) 733, 753, H) 713, and 745 nm for Pd_1_‐Cu_1_/b‐CN, probe delays under 365 nm excitation [70]. Reproduced with permission. Copyright 2023, Wiley‐VCH GmbH.

Light‐activable SANs leverage photoactive substrates to enhance catalytic activity by integrating an atomically dispersed metal active site with engineered light‐harvesting supporting materials. Upon light irradiation, photoexcited charge transfer and photothermal effects of synergistic mechanisms govern their performance enhancement. Semiconductor‐based supporting materials of photoexcited charge transfer (e.g., TiO_2_, g‐C_3_N_4_, carbon frameworks) absorb photons and generate electron‐hole pairs [60, 61, 62]. These carriers are effectively separated at the interface and migrate toward the single‐atom active site (Figure 2a) [63]. where they dynamically modulate electron density, local electron density state, and oxidation state. These changes strongly affect adsorption energies and catalytic selectivity, accelerating redox cycling and enhancing control over ROS production. In parallel, plasmonic nanomaterials (e.g., Au, Ag) act as efficient light absorbers, enabling localized enhancement of electromagnetic fields and promoting additional hot‐electron generation. These hot carriers transfer rapidly to the single‐atom metal active site, complementing the semiconductor‐driven pathway and expanding the overall light‐harvesting capability of the system [64]. For instance, anchored single‐atom Fe on semiconducting g‐C_3_N_4_, which enhanced light‐induced charge separation and led to improved photo‐Fenton activity. Using transient absorption (TA) spectroscopy with a probe at 573 nm, the authors explored the carrier kinetics in CN and Fe/D_c_‐CN. In Fe/D_c_‐CN, the initial picosecond component (τ1) is shortened relative to bulk CN, indicating accelerated exciton dissociation and rapid capture into shallow trap states. The subsequent tens to hundreds of picoseconds component (τ2) is redistributed, consistent with more facile migration into deeper traps and improved interfacial charge transfer, whereas the nanosecond component (τ3) is markedly prolonged, extending the effective lifetime of reactive carriers. These observations, together with suppressed steady state photoluminescence (PL), faster time‐resolved PL (TRPL) decay, enhanced transient photocurrent, and reduced interfacial charge transfer resistance, support a mechanism in which SA Fe sites reshape the shallow and deep trap energy landscape to suppress recombination and open additional extraction pathways (Figure 2b,c) [65].

In parallel, near‐infrared responsive supports provide a complementary photothermal effect, as plasmonic nanostructures, conjugated organic frameworks, and carbon materials efficiently convert absorbed photons into localized heat [66, 67]. This temperature elevation reduces activation energy barriers, accelerates interfacial reaction kinetics, and improves the flexibility of adsorption‐desorption dynamics at catalytic sites [68]. By modifying the local microenvironment, photothermal heating further facilitates optimal interaction between reactants and the single‐atom active site, leading to faster turnover rates and enhanced catalytic efficiency. Photoexcited charge transfer dynamically modulates the electronic state of the isolated metal active site, while photothermal heating simultaneously accelerates reaction kinetics [69]. In synergy, these processes achieve significantly higher conversion efficiencies and tunable ROS production compared to either mechanism alone. This integration of supporting materials engineering with atomic‐level coordination provides an effective and controllable platform for precision catalytic medicine. Through rational design of light‐activable SANs, it becomes possible to tailor electron transfer pathways, regulate ROS production, and realize external stimuli therapeutic functions under controlled optical stimuli.

#### 3.1.2 Single‐Atom Engineering for Selective Photocatalysis

While light‐harvesting supporting materials dictate how efficiently photons are captured and converted, the isolated metal active site determines how that harvested energy is utilized for catalysis. Upon photoexcitation, charge carriers generated in the supporting materials are transferred to the single‐atom active site, where local electronic structure, including oxidation state and electron density, is modulated, affecting absorption energies and catalytic selectivity. At this stage, the single‐atom metal active site functions as an electron sink or hole trapping site, which suppresses charge recombination and directs redox selectivity [70]. For example, in the case of Ag single‐atom doped on carbon nitride, the isolated Ag active site acts as charge separation regulators by suppressing electron‐hole recombination and serving as efficient hole‐trapping sites. This dual function not only prolongs the carrier lifetime but also directs the oxidation pathway, enabling the selective transformation of benzyl alcohol into benzaldehyde under light irradiation [71]. Similarly, island‐like single‐atom cobalt catalysts anchored on g‐C_3_N_4_ have been shown to modulate charge trapping and carrier lifetimes during light‐driven photo Fenton‐like reactions. The dispersed Co sites serve as localized traps that suppress rapid electron‐hole recombination and extend the availability of reactive carriers, thereby boosting •OH radical yield and enhancing pollutant degradation efficiency. Collectively, these findings demonstrate that precise engineering of the active site and coordination environment provides powerful control over charge‐trapping dynamics under light irradiation, resulting in higher catalytic efficiency [63]. Building on the active site role of single atoms, dual single‐atom architectures further partition charge management in time. Pd_1_‐Cu_1_/b‐CN features an in‐plane three‐coordinated Pd site and an interplanar four‐coordinated Cu site in layer‐layer‐stacked bulk‐like carbon nitride. These dual sites mediate rapid electron extraction and sufficient carrier retention for surface reactions (Figure 2d). Femtosecond TA at selected probe wavelengths (528, 753, and 713 nm) shows that the interplanar Cu‐N_4_ site exhibits a short τ in the picosecond regime, accelerating initial charge extraction, whereas the in‐plane Pd‐N_3_ site increases the contributions in the tens to hundreds of picoseconds and in the nanosecond regime, sustaining carrier retention and interfacial transport. Consequently, compared with pristine b‐CN or single metal doping, the TA signal persists longer, and TRPL reveals suppressed recombination and extended lifetimes (Figure 2e–h). The schematic summarizes these observations as a fast pathway (accelerated charge transfer mediated by Cu) and a slow pathway (long‐lived charge storage mediated by Pd), supporting a cascade mechanism in which the two single‐atom metal active sites operate sequentially to reduce charge losses while simultaneously increasing the density and residence time of reactive carriers. This optimization of charge kinetics improves access to active sites and interfacial charge transfer under photoexcitation, thereby significantly enhancing overall catalytic activity and efficiency [70].

Recent studies have further revealed that the valence electron number of the metal active site itself can decisively tune light‐driven pathways in single‐atom catalysts (SACs). In M_1_‐N_3_‐C_1_ sites (M = Mn, Fe, Co, Ni), it was demonstrated that metals with lower valence electron numbers, such as Mn, favor charge‐transfer‐dominated processes, leading to enhanced •O_2_^−^ generation and higher photothermal conversion efficiency by non‐radiative relaxation. In contrast, metals with higher valence electron numbers, such as Ni, promote energy‐transfer‐dominated pathways, which increase ^1^O_2_ yields while suppressing photothermal contributions. This work highlights that precise engineering of the metal center offers a powerful tool for balancing charge‐transfer versus excitonic contributions. Therefore, metal active sites are unable to control ROS generation, photothermal conversion, and reaction selectivity [72]. Collectively, these studies underscore that active‐site engineering serves as a central design principle for light‐activable SANs. At a general level, single‐atom metal active sites operate as electron sinks or hole‐trapping sites, effectively suppressing charge recombination and steering redox selectivity. And then the valence electron number of the active metal governs the balance between charge‐transfer and excitonic pathways, tuning both ROS type (•O_2_^−^ vs ^1^O_2_) and photothermal contributions. Taken together, these findings highlight that precise control over both the identity and coordination environment of single‐atom centers provides a powerful platform to regulate charge dynamics, ROS outputs, and catalytic selectivity under light irradiation.

### 3.2 Magnetic‐Activable SANs

#### 3.2.1 Supporting Material Engineering for Spin Polarization

**FIGURE 3:**
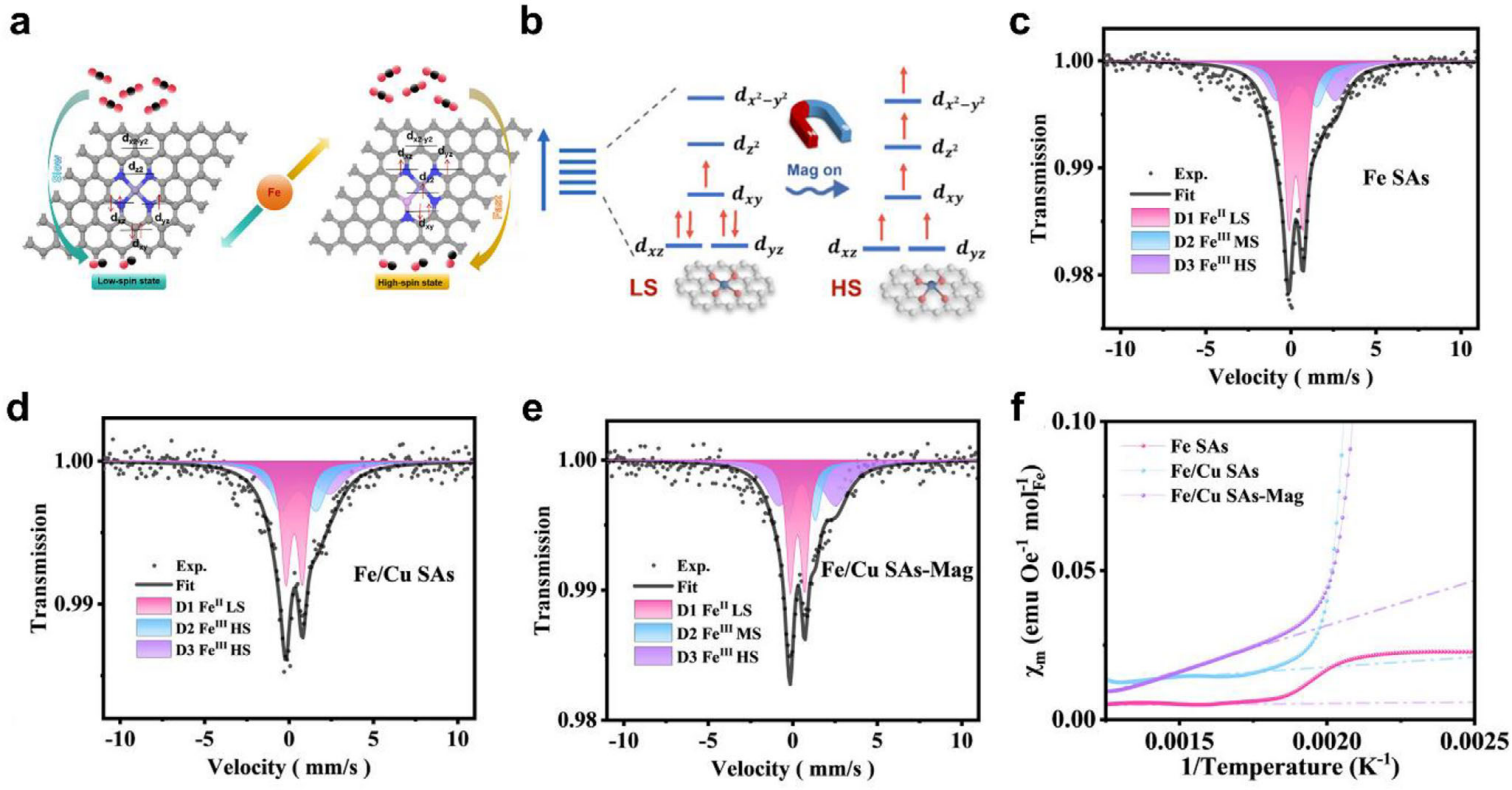
Magnetic‐activable SANs. (a) Schematic illustration of catalyst configuration (Left is Fe‐N‐C with a low‐spin state Fe(III) center, and right is Fe‐P‐N‐C with a high‐spin state Fe(III) center). Reproduced with permission [73]. Copyright 2025, Wiley‐VCH GmbH. (b) Schematic illustration of the transformation for the S@Fe‐NC electrode in a Zn‐S battery under an applied magnetic field. Reproduced with permission [79]. Copyright 2025, American Chemical Society. (c–e) Room‐temperature ^57^Fe Mössbauer spectra and f) temperature magnetic susceptibility curves of the Fe SAs, Fe/Cu SAs, and Fe/Cu SAs‐Mag. Reproduced with permission [80]. Copyright 2023, Wiley‐VCH GmbH.

Efficient magnetic activation requires two coordinated aspects: local active site tuning by defects and ligands, and supporting material‐mediated spin transfer pathways that enable collective alignment under an external field. Defect engineering perturbs the local coordination field. It can drive transitions from low spin to high spin and increase the polarization of unpaired electrons. This can promote spin‐selective exchange with triplet or paramagnetic intermediates and can lower adsorption and transition state free energies. A clear contrast appears at graphene defects. With an open vacancy defect (unpassivated defect), enhanced spin polarization and greater electronic availability around the single atom enable more favorable charge exchange and adsorption geometries, which lower barriers. With a nitrogen‐passivated defect, these benefits are attenuated, and the barriers rise [73, 74]. Ligand substitution provides a complementary handle. Replacing one N donor with P to form an N_3_P coordination can weaken ligand field splitting because M─P bonds are longer, 3p(P)‐3d(M) overlap is reduced, electronegativity is lower, while polarizability is higher, and local symmetry can be lowered. This tends to stabilize high‐spin configurations and, at the same time, fine‐tunes local charge distribution and Fermi‐level alignment (Figure 3a) [73]. As a result, the adsorption free energies of key intermediates and the potential‐determining step can shift to more favorable values, enabling higher activity and clearer pathway selectivity.

Magnetizable support becomes decisive once the spin and electronic preset is in place. MoS_2_ that hosts Ni single atoms at defective sulfur sites shows room temperature ferromagnetism through S p and Ni d hybridization and supporting material‐mediated exchange pathways. Applying a static magnetic field aligns spins, increases spin‐polarized transport, and markedly enhances water splitting performance. The improvement is reversible when the field is switched on and off. TiO_2_ with oxygen vacancies and Ti^3+^ centers builds exchange pathways that couple isolated Fe sites. Under a static field, spin‐polarized charge transport strengthens and nitrate to ammonia conversion improves in both selectivity and yield. The gains are again tunable with the field on and off. Across these connected spin networks, the external field promotes partial collective alignment and more efficient spin‐selective charge flow. This lowers effective barriers and accelerates the catalytic cycle. Changes in adsorption and electron transfer proceed in a spin‐dependent manner, consistent with the observed field‐responsive kinetics [75, 76]. Separately, magnetic supports such as Fe_3_O_4_ can, under alternating magnetic fields, generate magnetothermal and magnetomechanical stimuli, raising local temperature and mass transport. This can boost catalysis near single‐atom metal active sites and enable heating‐based biomedical applications [77].

In summary, effective magnetic activation couples two levers: tuning the local single‐atom site by defects and ligands, and building supporting material‐mediated spin transfer pathways that allow collective alignment under an external field. Defect creation and ligand substitution shift the spin state toward high spin, enhance spin polarization, and rebalance charge distribution and Fermi level, which lowers adsorption and transition barriers and clarifies pathway selectivity. After presetting the site, the support takes over. It routes spin‐polarized transport. A field aligns spins and enhances activity and selectivity.

#### 3.2.2 Single‐Atom Engineering for Magnetic Response

After engineering spin‐polarized transport through the supporting material, the single‐atom metal site becomes the primary control knob. Under magnetic stimuli, the responsiveness of the metal center, alongside supporting material‐mediated exchange and spin‐orbit effects, emerges as a key axis for tunable catalysis. Certain transition metal SANs (e.g., Fe, Co, Ru, Ni) can undergo magnetic field‐biased spin transitions (high spin ↔ low spin), which reorganize orbital occupancy and local electron density and thus alter reaction energetics. In this context, the single atom site and its first coordination shell act as the magnetically responsive unit. Unpaired spins that reside on hybrid metal‐ligand states couple to the applied magnetic field, producing Zeeman splitting and, when spin levels are near degenerate, field‐biased spin populations and local charge redistribution [78]. These changes tune the adsorption energies and pathways of spin‐dependent intermediates, enabling partially reversible control of catalytic performance under external magnetic fields.

In the case of Fe active sites, the partially filled 3d shell is sensitive to the balance between ligand field splitting and exchange. Applying a magnetic field produces Zeeman splitting of spin sublevels that can reweight spin populations and, under near‐degenerate conditions, can drive spin crossover. The field‐biased redistribution of spin density adjusts the local magnetic moment and tunes interactions with spin‐dependent intermediates and transition states, which can shift adsorption energies and reaction barriers and bias the preferred pathway. When supporting material‐mediated exchange and spin‐conserving transport are present, these effects are further amplified and can yield partially reversible changes in activity and selectivity under an external field. Fe SANs have been shown to exhibit spin‐state switching under alternating magnetic fields, enhancing electron transport and catalytic turnover in energy systems (Figure 3b) [79]. Similarly, adding a Cu neighbour to the Fe single atom site and applying an external magnetic field provides consistent evidence that Fe spin state and electronic structure are tunable by composition and field. First, the ^57^Fe Mössbauer spectra show that, from Fe single atoms to Fe/Cu co‐configured sites and then to Fe/Cu under an applied field, the fraction of low‐spin components decreases while the proportion of intermediate/high‐spin components increases (Figure 3c–e). The magnetic susceptibility plotted versus inverse temperature rises in the same order, supporting an increase in spin multiplicity and magnetic responsiveness (Figure 3f) [80]. Overall, the introduction of a Cu neighbor and the application of a magnetic field bias the spin population at the Fe site toward higher spin, and the associated charge redistribution and bond reorganization enhance the local magnetic moment and spin‐polarized charge transport, providing a physicochemical basis for changes in adsorption and pathway selection during catalysis. Ni single‐atom metal active site in relatively weak ligand field environments. For example, Ni coordinated by four nitrogen atoms can respond to an external magnetic field through Zeeman splitting. The field can reweight spin populations and modestly redistribute electron density at the Ni site, which stabilizes spin‐dependent intermediates while suppressing unfavorable side reactions. As a result, the preferred redox pathway is biased over competing routes, leading to improved catalytic selectivity and activity under suitable conditions [81]. By contrast, Ru single‐atom metal active site features expanded 4d orbitals and an intermediate ligand‐field strength, making them particularly sensitive to Zeeman splitting under external magnetic fields. This renders high‐spin ↔ low‐spin transitions more accessible, while redistributing electron density at the active site. As a result, the interaction with spin‐dependent intermediates (e.g., O_2_, ^*^OH, ^*^NH_2_) is optimized, lowering activation barriers and enhancing catalytic selectivity. Such magnetic responsiveness has been experimentally correlated with improved efficiency in multi‐electron reactions [82]. These findings, although primarily demonstrated in energy‐related catalysis, suggest that the same spin‐selective mechanisms could be readily extended to bio‐relevant redox pathways involving O_2_ and ROS intermediates. In practice, Fe single‐atom nano bowls exemplify how active‐site engineering can endow SANs with strong magnetic responsiveness for biomedical applications. The Fe^2+^/Fe^3+^ redox couple and partially filled 3d orbitals make the active site highly sensitive to external magnetic fields, where Zeeman splitting realigns spin states and redistributes electron density. These changes accelerate proton relaxation, improving magnetic resonance imaging (MRI) contrast and promoting the Fenton reaction, enhancing •OH generation and chemodynamic therapy (CDT) efficiency [83]. This case highlights how tailoring single‐atom active sites enables magnetically activable theranostics platforms that integrate both diagnostic and therapeutic functions.

### 3.3 Ultrasound‐Activable SANs

#### 3.3.1 Supporting Material Engineering for Ultrasound Response

**FIGURE 4:**
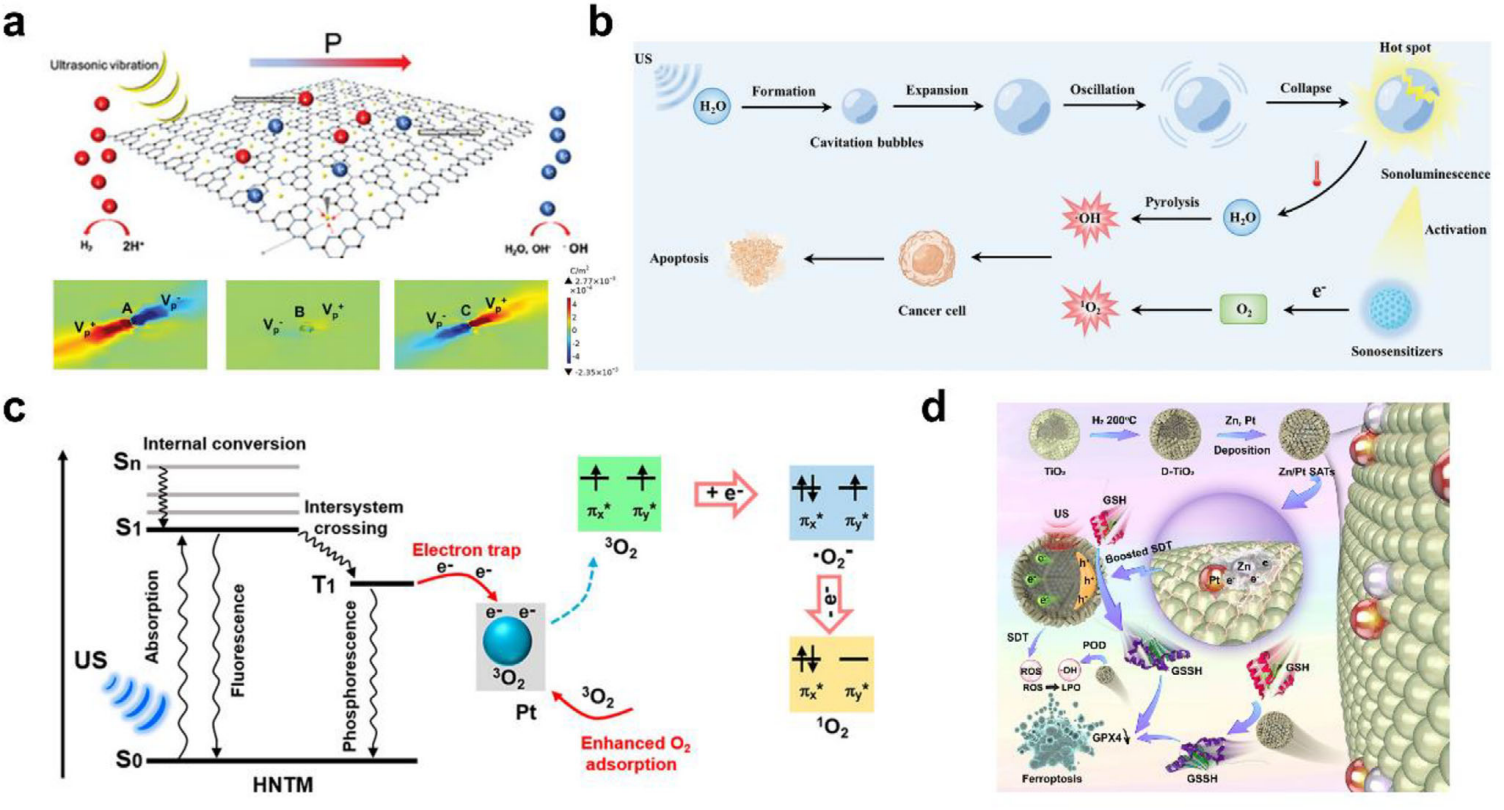
Ultrasound‐activable SANs. (a) Schematic illustration of the redox reaction of SA‐Pt/CN through piezo‐flexocatalysis and amplification of points A, B, and C for Pt anchored at SA‐Pt/CN. Reproduced with permission [84]. Copyright 2023, Wiley‐VCH GmbH. (b) Schematic illustration of the anticancer mechanisms of sonodynamic therapy (SDT). Reproduced with permission [86]. Copyright 2022, Wiley‐VCH GmbH. (C) Reactive oxygen species (ROS) generation mechanism of HNTM‐Pt under ultrasound. Reproduced with permission [87]. Copyright 2021, American Chemical Society. (d) Schematic illustration of Zn/Pt SATs sonosensitizer for cancer treatment. Reproduced with permission [152]. Copyright 2024, Springer Nature.

SANs inherently offer exceptionally high atomic utilization and reaction selectivity; however, their responsiveness to ultrasound is primarily dictated by the physicochemical properties of the supporting material. The supporting material absorbs energy from ultrasound. Thus, in ultrasound‐activable SANs systems, the supporting material functions not merely as a scaffold but as an energy transduction mediator, converting acoustic energy into electronic, thermal, and mechanical perturbations at the single‐atom active sites to enhance catalytic effect. A first pathway involves piezoelectric/flexoelectric charge induction: under ultrasound‐driven lattice deformation, suitable polar or semiconducting supporting materials (e.g., TiO_2_, ZnO, g‐C_3_N_4_, MoS_2_; in appropriate phases/defect states) can develop internal fields that modulate the electronic structure at M‐N_X_ sites and enhance electron and hole separation. For example, single‐atom Pt anchored on g‐C_3_N_4_ has been reported to leverage combined piezoelectric and flexoelectric responses under ultrasound, leading to polarization‐induced band modulation and markedly increased ROS production (Figure 4a) [84]. Under ultrasound, deformable supporting materials can also redistribute interfacial charge and transiently tune spin states at the SANs active site. These effects lower activation barriers and thereby accelerate ROS‐forming redox steps. In MoS_2_‐Zn SANs, such ultrasound‐induced lattice distortions have been shown to generate spin‐polarized electron flow toward Zn active sites, synergizing with transmetal charge transfer and enhancing ROS yield for targeted antibacterial therapy [85]. A second pathway is cavitation‐driven defect engineering: transient high‐pressure and high‐temperature microenvironments generated by microbubble collapse can restructure defect‐rich supporting materials (e.g., O‐deficient WO_3‐X_, N‐doped g‐C_3_N_4_), creating vacancies, shifting metal oxidation and spin states, and lowering activation barriers for ROS generation (Figure 4b) [86]. For example, anchoring metal single atoms onto oxygen‐vacancy‐rich WO_3‐X_ nanosheets enables cavitation‐induced defect reconfiguration and oxidation/spin‐state modulation to act synergistically, thereby facilitating O_2_ adsorption/activation and markedly increasing ROS generation; enhanced sonodynamic antitumor efficacy has subsequently been verified in both cellular and animal models [44]. Overall, these supporting materials‐mediated routes highlight supporting materials engineering as the primary lever for tuning the ultrasound responsiveness of SANs. Acoustic cavitation creates transient high‐pressure, high‐temperature microenvironments that restructure defects and lower activation barriers, while rational control of polarization, defect chemistry, and interfacial spin/charge dynamics enables high catalytic efficiency. The next section introduces engineering strategies for ultrasound‐activable single‐atom active sites.

#### 3.3.2 Single‐Atom Engineering for Ultrasound Catalytic Selectivity

The single‐atom active site of ultrasound‐activable SANs enables chemical reactivity and facilitates adsorption, electron transfer, and ROS generation. For instance, Fe single‐atom sites can act as catalytic hubs that capture ultrasound‐induced electrons and holes generated by cavitation and local excitation. They can slow recombination and promote charge transport. Fe‐N coordination can enhance the reactivity of O_2_ and H_2_O through metal‐ligand hybrid orbitals, thereby amplifying the production of ROS, including singlet oxygen, superoxide, and hydroperoxyl radicals. In the presence of H_2_O_2_ in the tumor microenvironment, the reversible Fe^2+^/Fe^3+^ cycle can drive Fenton‐like reactions to boost •OH generation, leading to concurrent enhancement of sonodynamic and chemodynamic therapy [56]. The coordination environment can regulate the oxidation state and spin distribution of Fe and thereby fine‐tune the type and ratio of ROS, while interactions with electron donors or defects can lower substrate activation barriers. Atomic‐level dispersion maximizes metal utilization and may reduce leaching risk, and strong coordination anchoring to graphitic C_3_N_4_ can stabilize the sites under repeated ultrasound exposure. Consequently, within the division of labor in sonodynamic therapy (SDT), where the support harvests energy and the active site sets chemical selectivity, Fe single‐atom sites function as key nodes that link charge separation, substrate activation, and ROS cascade reactions. In addition, Pt single‐atom nanozyme has been reported to play a complementary role. Under ultrasound, the sonosensitizer is excited from S_0_ to S_1_ and, after intersystem crossing, a long‐lived T_1_ state is formed. Pt single atom sites can trap excited electrons and delay their recombination while simultaneously strengthening O_2_ adsorption, creating a more favorable initial state. Two pathways then operate in parallel. First, the trapped electron is injected into the π^*^ orbital of adsorbed triplet O_2_ to produce superoxide (•O_2_^−^), the electron transfer route. Second, energy from T_1_ is transferred to O_2_ to generate singlet oxygen (^1^O_2_), the energy transfer route (Figure 4c) [87]. Owing to the heavy atom effect, Pt increases intersystem crossing efficiency, raises the T_1_ population, and thereby enhances the probability of ^1^O_2_ formation. Consequently, Pt single atom sites provide both charge collection and O_2_ adsorption/activation, systematically increasing the fluxes of •O_2_^−^ and ^1^O_2_ under ultrasound.

Moreover, Zn/Pt dual single‐atom sites operate under ultrasound with a clear division of labor and complementary synergy, thereby strengthening SDT and promoting ferroptosis. Conceptually, ultrasound generates charge carriers in the support through cavitation and local heating, while Pt single‐atom sites provide an electronic structure favorable for O_2_ activation and the conversion of peroxide species, thereby guiding ROS generation. In contrast, Zn single‐atom sites fine‐tune local electron density, band edges, and the coordination environment, and they facilitate intermetal charge transfer, which suppresses electron–hole recombination and assists charge delivery to Pt sites (Figure 4d). This dual‐site synergy increases ROS flux under ultrasound and leads to lipid peroxidation, with enhanced ferroptosis markers such as GPX4 downregulation, elevated lipid ROS. In summary, the Pt active site serves as the chemical activation center for oxygen and peroxide species, whereas the Zn active site acts as a coordinator of charge, coordination, and interfacial transport. Overall, the two single‐atom active sites convert ultrasound‐derived energy into ROS pathways more efficiently, suggesting high catalytic performance and therapeutic response in tumor environments. Thus, single‐atom active sites are increasingly recognized not only as structural motifs but as tunable catalytic platforms where atomic‐level control over metal identity, coordination geometry, and redox dynamics can significantly enhance ultrasound‐driven catalytic activity. By precisely engineering these parameters, it becomes possible to optimize energy transfer efficiency, regulate ROS generation pathways, and tailor reaction selectivity under sonochemical conditions. Such advancements in active‐site design pave the way for more efficient and controllable catalytic systems in ultrasound‐mediated applications.

### 3.4 Other Stimuli‐Activable SANs

Other external stimuli can also regulate nanozyme activity, including temperature, electric fields, microwaves, and X‐rays. Thermal stimuli are rarely applied alone but are typically integrated with other external stimuli, such as photothermal or magnetothermal effects, to modulate catalytic activity through localized heating and enhanced reaction kinetics [88]. Electric fields can directly regulate the electronic structure of nanozymes and facilitate electron transfer processes. In particular, recent studies have shown that electrical stimulation can modulate the d‐band center and electron density of single‐atom active sites, thereby enhancing substrate adsorption and catalytic activity. Moreover, electric‐field‐driven electrochemical processes have been utilized in certain biosensing systems, where catalytic reactions are coupled with measurable current signals for real‐time detection. However, studies on electrically stimulated nanozyme systems remain scarce [17, 89]. Microwave irradiation provides rapid and volumetric energy input by inducing dipole polarization and charge oscillation within materials, thereby enhancing catalytic activity through accelerated reaction kinetics and promoted electron dynamics [90]. However, microwave‐responsive nanozyme systems are still relatively underexplored, with only a limited number of studies reported to date, particularly for single‐atom nanozymes [91, 92]. Despite this, rational design of substrate materials (e.g., high dielectric loss, abundant defect sites, and conductive frameworks) is expected to enable effective microwave activation of single‐atom nanozyme systems. In contrast, X‐ray irradiation has already been demonstrated to activate single‐atom nanozymes through radiation‐induced processes, including radiolysis and the generation of reactive species (e.g., H_2_O_2_ and ROS), which can further participate in cascade catalytic reactions [16]. In addition, X‐ray irradiation offers significantly deeper tissue penetration than NIR light and is not strictly limited by bandgap constraints, enabling effective activation in deep or optically inaccessible biological environments [93]. However, such studies remain relatively limited compared to conventional light‐driven systems. Overall, although these external‐stimulus strategies remain relatively underexplored with few reported studies, they demonstrate significant potential for advancing nanozyme design and expanding their applications.

### 3.5 Multi‐Stimuli‐Activable SANs

Multi‐stimuli‐activable SANs have recently emerged as an advanced strategy for enhancing catalytic controllability and therapeutic performance. Although research in this area is still at an early stage, several representative examples have demonstrated the feasibility and potential of integrating multiple external stimuli within a single SAN platform. For instance, photo‐activable SAN systems combined with magnetic resonance imaging (MRI) functionality have been reported. In Fe‐based single‐atom nanobowl systems, near‐infrared (NIR) irradiation enhances Fenton‐like catalytic activity, thereby improving chemodynamic therapy efficiency, while magnetic properties enable MRI‐based imaging [83]. Similarly, in self‐assembled Ru single‐atom nanozyme systems, light irradiation activates photodynamic therapy (PDT), whereas Mn‐based components provide MRI functionality, enabling imaging‐guided therapeutic processes [94]. In addition, although not exclusive to SANs, recent studies have demonstrated that nanozyme systems integrating multiple external stimuli, such as magnetic targeting, ultrasound‐triggered catalytic activation, and biochemical cascade reactions, can cooperatively regulate spatial localization, reaction activation, and substrate supply [95]. These findings highlight the feasibility of multi‐stimuli integration and suggest that such strategies can be extended to SAN systems. From a design perspective, the synthesis of multi‐stimuli‐activable SAN systems does not fundamentally differ from conventional SANs. Instead, the key lies in the rational selection and integration of stimulus‐activable components. In particular, the selection of materials capable of responding to multiple external stimuli is crucial, as it enables coordinated and synergistic activation under different conditions. This allows each stimulus to trigger specific functions within the system. Overall, multi‐stimuli integration represents a promising direction for advancing nanozyme design. Future efforts should focus on the rational combination of external stimuli and material systems to achieve more precise catalytic regulation and expanded functionality. With further development, multi‐stimuli‐activable SANs are expected to play an important role in next‐generation theranostics applications.

## 4 Synthesis of SANs

The catalytic performance of a single metal atom, such as SACs, often surpasses that of bulk nanocatalysts, and the extended reaction surface area provides additional benefits [96]. The atomic‐level design of SACs still faces several challenges. The catalytic activity, electrical conductivity, redox stability, and overall stability of the single atom are highly dependent on the properties of the support material used to anchor the single atom [97]. Thus, the choice of the support material is often dictated by the specific application, and the synthesis method is consequently tailored to the desired catalytic reaction site [98, 99]. In the same sense, the synthesis of SANs can leverage the strengths of various SACs synthesis methods. In this chapter, we will explore synthetic techniques in overcoming challenges to enhance catalytic activity, stability, and characteristic properties for different applications [100, 101].

### 4.1 Bottom‐Up Synthesis Methods

The bottom‐up approach involves using atomic or molecular precursors to sequentially assemble single‐atom structures on a substrate [102]. The characteristics of bottom‐up synthesis methods are precise control. It is allowing for fine‐tuning of the coordination environment and dispersion of the active atoms [103, 104]. It is possible to make a high dispersion active site that theoretically enables up to 100% atom utilization efficiency to maximize catalytic activity and reactivity [105].

#### 4.1.1 Atomic Layer Deposition for SANs and Metal Support

**FIGURE 5:**
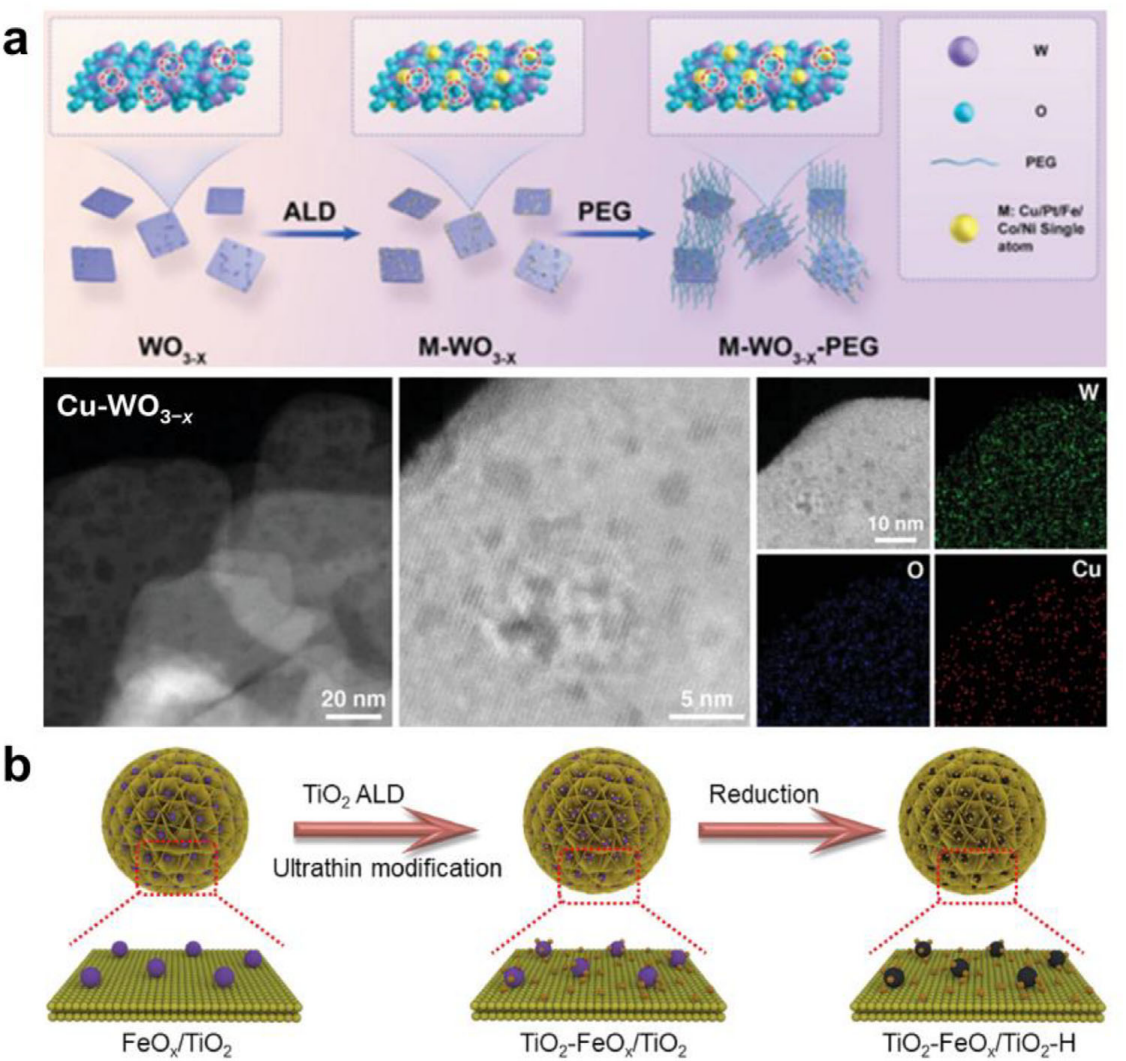
Bottom‐up synthesis. (a) Atomic layer deposition of different metal single‐atoms into WO_3‐x_ nanosheets to form M‐WO_3‐x_. High‐angle annular dark‐field scanning transmission electron microscopy (HAADF‐STEM) images and the energy‐dispersive X‐ray spectroscopy (EDX) elemental mapping images of Cu‐WO_3‐x_ nanosheets [44]. Reproduced with permission. Copyright 2024, Wiley‐VCH GmbH. (b) Atomic layer deposition of metal support TiO_2_ onto FeO_x_/TiO_2_ to form TiO_2_‐FeO_x_/TiO_2_ by ultrathin modification strategy [109]. Reproduced with permission. Copyright 2022, American Chemical Society.

Atomic layer deposition (ALD) is a precise synthesis technique that deposits thin films on a substrate in an atomic‐layer‐by‐atomic‐layer manner via sequential, self‐limiting surface reactions of gaseous precursors [106]. In the synthesis of SANs, ALD is utilized to anchor metal atoms on the substrate surface and individually and precisely control their coordination environment [107]. ALD proceeds by sequentially introducing two or more volatile precursors into the chamber, with an intervening inert gas purging step to remove the residues of the preceding precursor completely [108]. Zhou et al. introduced metal single atoms, mainly Cu with Pt, Fe, Co, Ni as comparisons, onto defect‐rich WO_3‐x_ nanosheet supports by atomic layer deposition ALD to enhance sonodynamic therapy SDT activity (Figure 5a) [44]. The synthesis heats WO_3_·H_2_O nanosheets in air to obtain defect‐poor WO_3_ and in N_2_ to obtain oxygen vacancy‐rich WO_3‐x_. ALD with only a few cycles anchors single atoms at active sites, such as oxygen vacancies or OH groups, and suppresses aggregation. The resulting SA‐OV interface promotes charge separation and transport under ultrasound and markedly increases ROS generation, for example, ^1^O_2_ and •O_2_^−^, giving higher SDT activity than the same single atoms on defect‐poor WO_3_. HAADF‐STEM 20 nm and high magnification 5 nm images show dotted contrast from isolated metal atoms without aggregated particles. STEM EDS maps show uniform W and O with dotted Cu distribution, confirming single‐atom dispersion. Overall tuning defect density, oxygen vacancies, ALD cycle number, and metal identity control single‐atom dispersion coordination environment and interfacial electronic structure, yielding an efficient single‐atom nanozyme operable at low ultrasound power. Building on this, Qin et al. applied only a small number of ALD cycles to high surface area TiO_2_ nanoflowers to achieve predominantly single atom dispersion of Fe on FeO_x_/TiO_2_ (as prepared), while the co‐formed ultrasmall FeO_x_ clusters served as electron transfer auxiliaries, thereby enhancing POD‐like activity (Figure 5b) [109]. They then deposited one additional TiO_2_ cycle to gently cap the active sites, yielding TiO_2_‐FeO_x_/TiO_2_ (ALD coated), which pinned the sites and finely tuned the metal support interaction. Finally, a mild reduction afforded TiO_2_‐FeO_x_/TiO_2_‐H (H reduced), adjusting the oxygen vacancy and Ti^3+^ levels in TiO_2_ and the overall charge density to optimize reactivity. Taken together, by controlling the ALD deposition cycle number, introducing an ultrathin TiO_2_ coating, and performing a post‐reduction step, they simultaneously fine‐tuned the single atom/nanocluster ratio, local coordination environment, and interfacial electronic structure, thereby reporting SANs optimized for POD‐like reactions.

#### 4.1.2 Chemical Precipitation Coordination of Metal Into Supporting Frameworks

**FIGURE 6:**
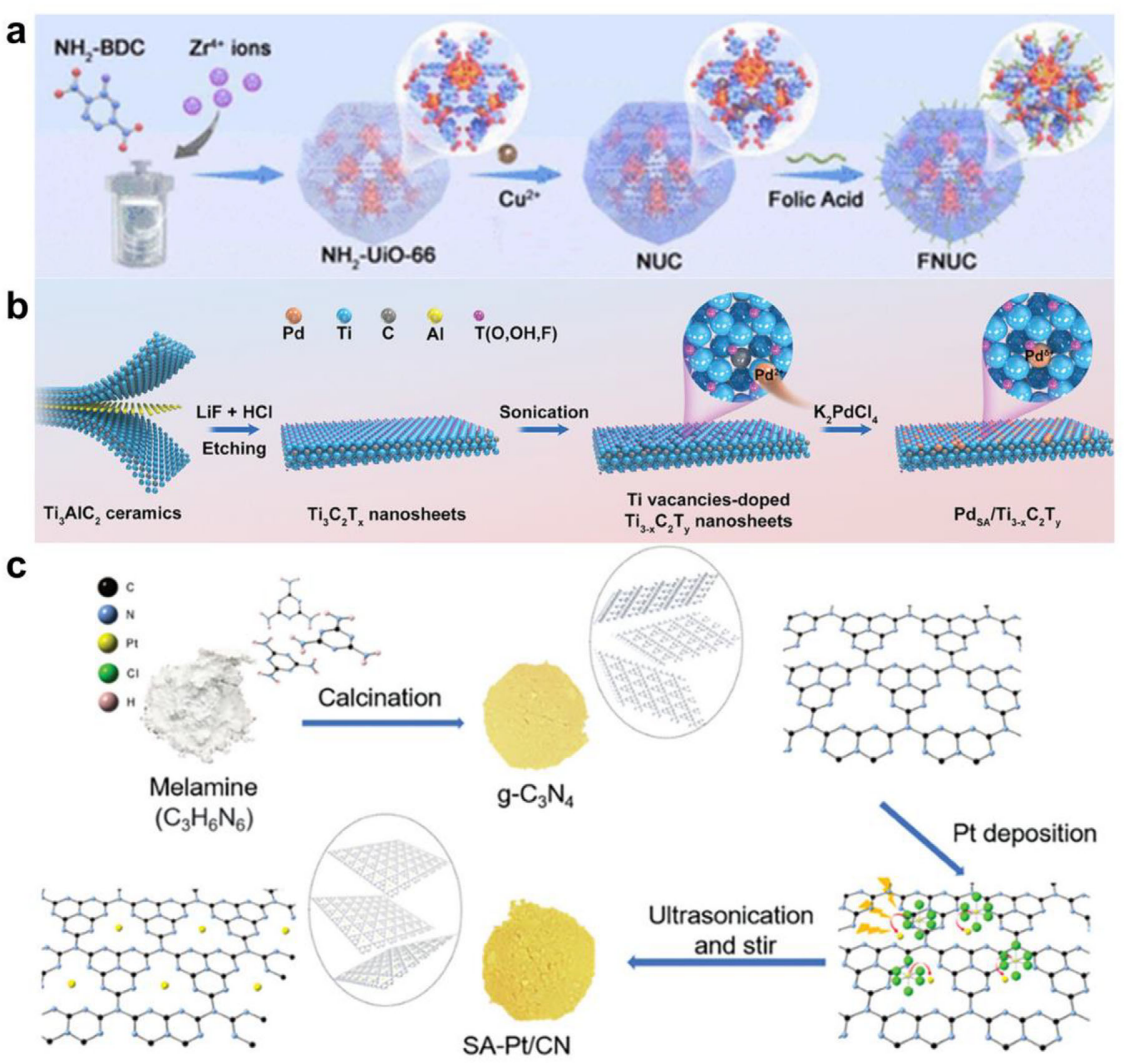
Bottom‐up synthesis. (a) Chemical coordination of Cu single atoms into NH_2_/‐COO^−^ or defect sites of NH_2_‐UiO‐66 to form FNUC. Reproduced with permission [116]. Copyright 2024, American Chemical Society. (b) Chemical coordination of Pd single atoms into Ti vacancies of Ti_3_C_2_T_x_ nanosheets to form the PdSA/Ti_3‐x_C_2_T_y_ SAzyme. Reproduced with permission [117]. Copyright 2024 Wiley‐VCH GmbH. (c) Chemical coordination of Pt single atoms into N donor and defect sites in g‐C_3_N_4_ to form the SA‐Pt/CN. Reproduced with permission [84]. Copyright 2023 Wiley‐VCH GmbH.

The Chemical Precipitation/Adsorption method is one of the most traditional and widely used bottom‐up approaches for synthesizing SACs. This method aims to efficiently disperse and anchor metal atom precursors from the liquid phase onto the support surface [110]. For example, Cu^2+^ precursors (e.g., Cu(NO_3_)_2_) are introduced into a suspension of zeolitic imidazolate framework‐8 (ZIF‐8), where they coordinate to nitrogen donor sites of the imidazolate framework or to surface functional groups. Subsequent gentle reduction or thermal activation removes labile ligands and fixes isolated Cu centers as Cu‐N_x_/O_x_ single‐atom sites while suppressing nanoparticle nucleation [111, 112, 113, 114, 115]. Wang et al. reported the synthesis of an ultrasound‐enhanced nanozyme by anchoring Cu single atoms at coordination sites within a UiO‐66‐type MOF and activating them with ultrasound to simultaneously promote ROS generation and GSH depletion. The synthesis proceeds by first preparing UiO‐66‐NH_2_ with regular porosity from 2‐Aminoterephthalic acid (NH_2_‐BDC) and Zr^4+^, then introducing Cu^2+^ in solution to coordinate at ‐NH_2_/‐COO^−^ or defect sites within the framework to obtain NUC with isolated Cu single atoms without aggregation, and finally coupling folic acid to surface ‐NH_2_ groups to yield Cu‐based single‐atom Sazymes (FNUC) with targeting capability toward FA‐receptor‐overexpressing cancer cells (Figure 6a). The N/O donor sites and defects of UiO‐66‐NH_2_ stabilize Cu^2+^ as single atoms and suppress aggregation, while the anchored Cu centers catalyze ROS formation via the Cu^2+^/Cu^+^ redox cycle and concomitantly consume intracellular GSH, weakening antioxidant defenses. Consequently, under ultrasound irradiation, FNUC shows markedly increased ROS levels and cell death in vitro, and effective tumor growth inhibition in vivo, demonstrating the synergy between single‐atom MOFs and ultrasound activation [116]. Geng et al. reported a synthesis strategy that first creates “vacancies (anchor sites)” on the surface and then stabilizes Pd single atoms by coordinating/adsorbing them at those sites (Figure 6b). Specifically, the surface terminations and Ti vacancies of Ti_3_C_2_T_γ_ᵧ nanosheets are adjusted to increase high‐activity sites exposing oxygen, hydroxyl, and carbide carbon. A diluted Pd(II) solution is then introduced so that Pd‐C‐centered single‐atom sites form at these positions, while low precursor concentration and short treatment time suppress nucleation and surface migration. In the final step, residual ligands are removed, and atomic diffusion is further hindered to lock the single‐atom state. In summary, by increasing surface vacancies, applying low‐concentration coordination‐adsorption, and finishing with mild activation, the authors achieved highly dispersed Pd single‐atom sites and concurrently aligned the interfacial electronic structure between support and metal. Under ultrasound, the resulting SACs rapidly consume GSH and promote the activation of endogenous H_2_O_2_ and oxygen, thereby lowering both hypoxia and antioxidant barriers. As a result, SDT‐induced ROS signaling remodels the tumor microenvironment toward immune activation, yielding a pronounced sono‐immunotherapy synergy [117]. Wang et al. reported an adsorption‐based wet‐chemistry route that uses N donors and defect sites in g‐C_3_N_4_ as anchors (Figure 6c). Melamine was thermally polymerized at 650°C to make g‐C_3_N_4_. The powder was dispersed in a water and methanol mixture. A dilute H_2_PtCl_6_ solution was added dropwise to coordinate the Pt precursor on pyridinic or amino N and defect sites. The mixture was then photoreduced. Photoelectrons converted Pt(IV) to Pt‐N single‐atom sites. Low precursor concentration, short irradiation, low ionic strength, and controlled pH suppressed nucleation and surface migration, which prevented nanoparticle growth. The resulting Pt single atoms formed strong M‐N coordination with g‐C_3_N_4_ and acted as charge‐trap and transfer hubs. Under ultrasound, polarization charges accumulated at these Pt sites and accelerated reduction and oxidation reactions [84].

#### 4.1.3 Pyrolysis to Form Metal‐Anchored Carbon Support

**FIGURE 7:**
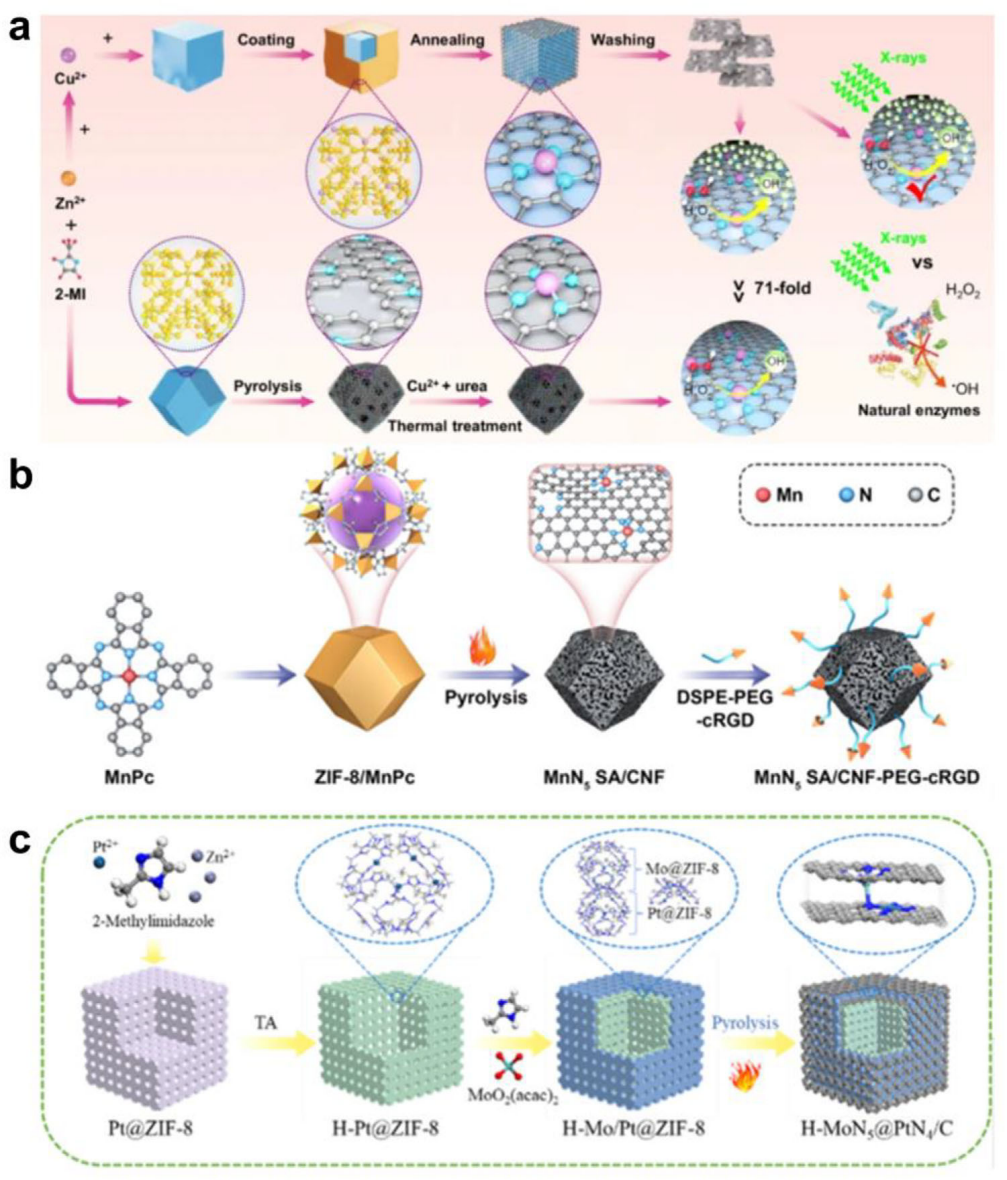
Bottom‐up synthesis. Pyrolysis of metal precursors and supporting frameworks for (a) CuN_3_‐SAzyme and CuN_4_‐SAzyme, (b) MnN_5_ SA/CNF, and (c) H‐MoN_5_@PtN_4_/C. (a) Reproduced with permission [93]. Copyright 2024, Springer Nature. (b) Schematic Illustration of Synthesis Process. Reproduced with permission [124]. Copyright 2024, American Chemical Society. (c) Scheme of the synthetic procedure of H‐MoN_5_@PtN_4_/C. Reproduced with permission [125]. Copyright 2024, American Chemical Society.

This route can serve as both a primary synthesis and a post‐treatment. In a typical pyrolytic synthesis, metal precursors are mixed with a carbon precursor (or other supporting material) and then heated at high temperature under reducing, vacuum, or inert atmospheres (often N_2_, Ar, H_2_, or NH_3_ when N‐doping is desired) [118, 119]. During pyrolysis, organic ligands carbonize and anchoring sites (e.g., M‐N_4_, M‐C_x_, vacancy sites) form in situ, enabling the fixation of isolated metal atoms while suppressing sintering [120, 121, 122, 123]. Wu et al. reported a single‐atom copper nanozyme in which tuning the local coordination boosts both radiation resistance and peroxidase‐like activity. In the CuN_3_ route, a Cu‐organic layer is coated on ZIF‐8 and annealed to carbonize into N‐doped carbon, fixing Cu as Cu‐N_x_ single sites; subsequent washing removes Zn and leaves Cu single atoms on a porous carbon shell. In the CuN_4_ route, ZIF‐8 is first pyrolyzed to N‐doped carbon polyhedra, then Cu^2+^/urea is adsorbed and thermally treated so that urea provides N to form Cu‐N_x_ single sites (Figure 7a). In both routes, the key is converting ZIF‐8 into an N‐doped carbon support and stabilizing Cu single atoms, with the coat, annealing, and wash path offering better electron transfer and site accessibility and thus higher activity. By constructing an unsaturated CuN_3_ single site with a coordination number of three, H_2_O_2_ activation is optimized, and strong •OH generation is maintained under radiation without performance loss [93]. Yuan et al. reported a nanozyme that embeds Mn single sites (MnN_5_) by pyrolyzing a ZIF‐8 template combined with manganese phthalocyanine (MnPc). A ZIF‐8/MnPc composite was first prepared and thermally treated to carbonize the framework and volatilize Zn, yielding a porous carbon nanoframe (CNF) while reconstructing the Mn‐N motif to form MnN_5_ single‐atom sites inside the frame (MnN_5_ SA/CNF). The surface was then functionalized with DSPE‐PEG‐cRGD to improve blood stability and in vivo circulation and to endow targeting toward αvβ3‐integrin‐overexpressing tumors (Figure 7b). The MnN_5_ sites, together with the polarization/radical environment generated by ultrasound cavitation and the electron pathways of the defect‐rich carbon frame, sustain ROS generation even under oxygen‐poor conditions. Consequently, consistent antitumor efficacy was achieved in an orthotopic breast cancer model with a hypoxic TME, establishing a design principle for oxygen‐independent sonodynamic therapy [124]. Zhao et al. reported a nanozyme in which M‐N_4_X single atom sites are embedded by precoordination of metal and axial ligand precursors to a support, followed by pyrolysis (Figure 7c). First, Pt^2+^ was co‐introduced during ZIF‐8 assembly (2‐methylimidazole/Zn^2+^) to obtain Pt@ZIF‐8 with Pt trapped inside the framework. Tannic acid treatment converted it into a hollow architecture (H‐Pt@ZIF‐8) that preserved the outer scaffold and created a thin shell and inner void. MoO_2_(acac)_2_ was then adsorbed to place Mo precursors uniformly on the shell. In the key pyrolysis step, ZIF‐8 carbonized into N‐doped carbon, and Zn volatilized, while Pt and Mo precursors reorganized and were fixed as single‐atom sites, PtN_4_ in the core and MoN_5_ in the shell. The resulting thin shell hollow carbon cube (H‐MoN_5_@PtN_4_/C) showed improved mass and electron transport with spatially separated dual single atom centers. Axial ligand‐induced d orbital splitting rearranged spin and electronic structures, weakened destructive d and π conjugation, and optimized adsorption energies, yielding high POD‐ and OXD‐like activities with strong ROS generation [125].

#### 4.1.4 Coordination‐Derived Self‐Assembly of Precursors

**FIGURE 8:**
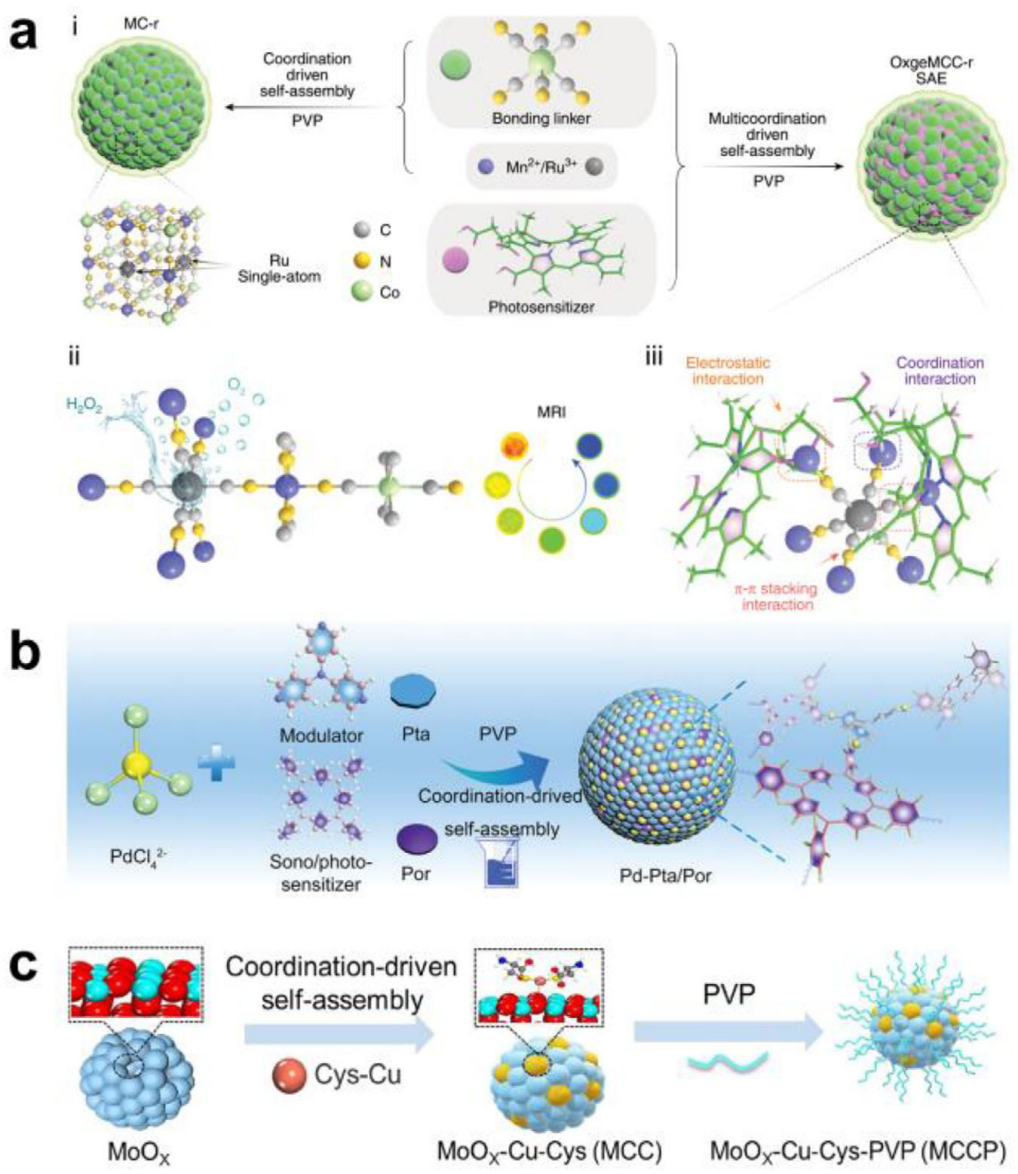
Bottom‐up synthesis. Coordination‐derived self‐assembly of (a) OxgeMCC‐r, (b) Pd‐Pta/Por, and (c) MoOx‐Cu‐Cys‐PVP (MCCP). (a) OxgeMCC‐r consists of a catalytically active single‐atom Ru site anchored in MCC with an outer PVP protection layer (i). Partial molecular structure of OxgeMCC‐r with active single‐atom Ru site serving as catalase‐like nanozyme for oxygen generation (ii). Multicomponent coordination interactions within the OxgeMCC‐r SAE (iii) [94]. Reproduced with permission. Copyright 2020, Springer Nature. (b) The amplified image is the chemical structure of Pd‐Pta/Por [126]. Reproduced with permission. Copyright 2021 Wiley‐VCH GmbH. (c) High‐loading Cu single atoms MCCP Sazymes [127]. Reproduced with permission. Copyright 2023, American Chemical Society.

Self‐assembly is a spontaneous ordering process where small molecules organize into well‐defined architectures via noncovalent forces such as hydrogen bonding, van der Waals interactions, pi‐pi stacking, charge transfer, and donor‐acceptor pairing. Compared with multistep conventional syntheses, this route offers simpler workflows and tighter structural control. In particular, the directional metal ligand coordination inherent to self‐assembly is well suited to building SACs with uniform local coordination and well‐defined active sites. Wang et al. reported the synthesis of a nanozyme (OxgeMCC‐r) by embedding Ru single sites into the lattice of a Prussian blue‐like MOF (Mn_3_[Co(CN)_6_]_2_) through partial substitution of Co with Ru^3+^, followed by coordination‐driven self‐assembly with PVP and Ce6 (Figure 8a) [94]. In solution, Ru^3+^ was first introduced to generate Ru single sites in the framework, then PVP as a linking polymer and Ce6 as a photosensitizer were added to achieve particle formation in a single pot. Coordination among CN ligands and metal ions with PVP and Ce6, together with *π*–*π* stacking of Ce6 and electrostatic attraction, produced colloidal particles with stable dispersion of Ru single atoms. By adjusting the ratios of PVP and metal ligands, the particle size and Ce6 loading were controlled, and a highly dispersed single‐atom MOF was obtained under aqueous conditions without additional heat treatment. OxgeMCC‐r catalyzed the conversion of endogenous H_2_O_2_ to O_2_, alleviated tumor hypoxia, and consequently enhanced singlet oxygen generation and antitumor efficacy in photodynamic therapy [126]. Du et al. reported a Pd‐Pta/Por nanozyme in which Pd single sites are embedded via coordination‐driven self‐assembly using a PdCl_4_^2−^ precursor, 5,10,15,20‐tetra(4‐pyridyl)porphyrin (Por), and tri(pyridin‐4‐yl)amine (Pta) (Figure 8b). In solution, Por (photo/sono sensitizer), Pta (structure‐modulating ligand), and PVP (stabilizer/coordination helper) are mixed, then PdCl_4_^2−^ is added to trigger self‐assembly, yielding spherical colloids where Pd is coordinated to pyridinic N and fixed as highly dispersed single sites. In the product, Por harvests light/ultrasound energy to generate ROS, while Pd single sites promote electron transfer and peroxidative reactions, delivering clear synergy in chemicatalytic, sonodynamic, and photodynamic (trimodal) tumor therapy. Zhou et al. reported a coordination‐driven self‐assembly that embeds Cu single atoms on MoO_x_ nanoparticles and stabilizes them with a PVP shell to form MoO_x_‐Cu‐Cys‐PVP nanozymes (Figure 8c). The SH, NH_2_, and COO^−^ donor groups of L‐cysteine chelate Cu to give Cu‐N/S sites, while the carboxylate anchors on MoO_x_ surface defects or ‐OH groups, yielding MoO_x_‐Cu‐Cys (MCC) with atomically dispersed Cu. Subsequent PVP coating affords MoO_x_‐Cu‐Cys‐PVP (MCCP) with improved colloidal and in blood stability. MCCP reaches a Cu single‐atom loading of 10.10 wt.% and, via cysteine‐chelated Cu single atoms (Cu‐N/S) featuring interfacial Cu‐O coordination on MoO_x_, accelerates electron transfer and delivers catalase‐like H_2_O_2_ decomposition activity 138 fold higher than MnO_2_ nanozymes with 14.3 fold higher substrate affinity than natural catalase. The Cu single atoms also drive Fenton‐like •OH generation, and under X‐ray irradiation, MCCP converts O_2_ to ^1^O_2_ (radiodynamic therapy), producing multiple ROS that enhance radiosensitization and significantly suppress tumor growth [127].

### 4.2 Top‐Down Synthesis Methods

The top‐down approach involves generating single atoms by removing unwanted portions or dispersing metal from existing bulk or nanoparticle materials [128]. It is attractive for scale‐up because workflows are relatively simple compared with bottom‐up routes, enabling high‐throughput and lower cost manufacturing [129, 130]. However, precise control over the active site is more difficult since converting nanoparticles to isolated atoms can introduce unintended etching and oxidation, reduce atomic utilization, and complicate complete dispersion and long‐term stabilization of single atoms. Despite these challenges, its lower synthetic complexity and strong compatibility with mass production make top‐down synthesis a promising path toward commercialization [131].

#### 4.2.1 Chemical Etching of Metal Support for Single‐Atom Deposition

**FIGURE 9:**
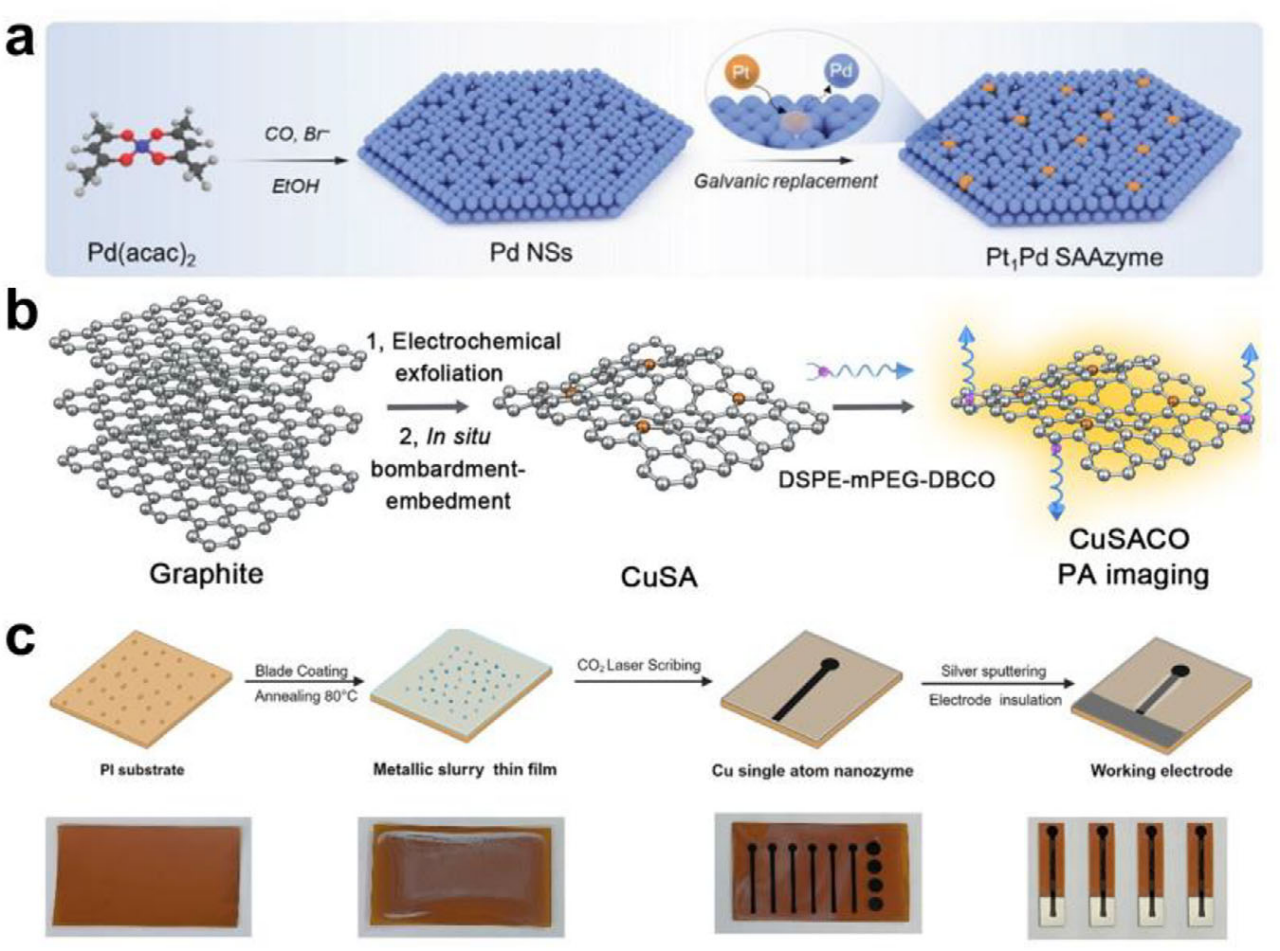
Top‐down synthesis. (a) Galvanic replacement via chemical etching of Pd support and deposition of Pt single atoms for the Pt_1_Pd SAAzyme [42]. Reproduced with permission.Copyright 2023 Wiley‐VCH GmbH. (b) High‐energy dispersion of Cu plasma to form CuSA into graphene nanosheets. CuSA was prepared by an electrochemical exfoliation and in situ bombarding‐embedding strategy, and CuSACO was prepared via modification of CuSA using DSPE‐mPEG2000‐DBCO [43]. Reproduced with permission. Copyright 2024, Wiley‐VCH GmbH. (c) High‐energy dispersion of CO_2_ laser to immobilize Cu single atoms into carbon support (CuSAN) [136]. Schematic representation of the synthesis pathway. Reproduced with permission. Copyright 2024, Wiley‐VCH GmbH.

The chemical etching/oxidation method is a top‐down route to SACs in which surface or lattice atoms are selectively removed from preformed metal nanoparticles or bulk structures to achieve atomic dispersion [129, 130]. In the etching pathway, acids, halides, or complexing ligands strip surface atoms into solution, and the liberated ions are re‐anchored by chemisorption/coordination onto defect‐rich supports to form isolated single sites. In the oxidation pathway, surface atoms are oxidized to ionic species, lowering cohesive energy and enabling detachment, followed by stabilization at heteroatom‐ or defect‐anchoring motifs on the support [132, 133]. Li et al. reported a Pt_1_‐Pd single‐atom alloy (SAA) nanozyme obtained by applying galvanic replacement‐based chemical etching to Pd nanosheets (Figure 9a). Surface Pd was selectively leached and replaced in situ by isolated Pt atoms, which were then immobilized in the Pd lattice. Driven by the redox‐potential difference (Pd leaves while Pt single atoms enter), this top‐down chemical etching/oxidation process allows precise control of replacement depth and Pt single‐atom density by tuning precursor concentration, halide ligands (Cl^−^/Br^−^), pH, temperature, and reaction time. The resulting Pt atoms are fixed as dispersed, active single sites that optimize charge transfer and intermediate binding; suppressing over‐etching and re‐deposition preserves the ultrathin morphology and high surface single‐atom dispersion characteristic of the SAA nanozyme. Functionally, the Pt_1_Pd SAA balances the binding energies of key intermediates across POD/CAT/GPx‐like pathways, accelerating H_2_O_2_ decomposition and O_2_ activation and often depleting GSH to weaken cellular antioxidant defenses. Under light irradiation, photothermal/photocatalytic effects of the support synergize with SAA‐mediated electron transfer to amplify ROS generation, leading to pronounced cancer cell apoptosis and suppressed tumor growth [42].

#### 4.2.2 High‐Energy Dispersion for the Single Atom Deposition

High‐energy dispersion is a methodology for synthesizing SACs, primarily categorized as a top‐down approach. This method is characterized by forcing the dispersion of metal atoms via a kinetic route rather than thermodynamic equilibrium [134, 135]. These methods utilize a high‐energy source (e.g., laser, plasma, thermal) to decompose the atoms of a large metal structure like NP and subsequently capture them onto a support, thereby stabilizing the single‐atom state. These methods can be categorized based on the energy source, including laser/plasma ablation, strong thermal shock, and high‐energy ball milling.

##### 4.2.2.1 Laser/Plasma‐Assist

This method utilizes high‐intensity energy (a laser pulse or plasma) irradiated onto a solid or liquid target material, causing the surface material to be explosively ejected as plasma, a phenomenon known as ablation. This process forcibly separates the atoms from the metal target or precursor and subsequently anchors them onto a support to synthesize SACs [128, 129, 130, 131, 132]. Wu et al. reported a single‐atom nanozyme synthesis in which Cu single atoms are inserted and fixed in situ into defect sites of graphene using high‐energy metal plasma (Figure 9b). Graphite is first electrochemically exfoliated to obtain defect‐rich graphene, then metal plasma generated by magnetron sputtering bombards and embeds Cu atoms into vacancies/defects to yield Cu single atom nanozyme (CuSACO). The surface is subsequently modified with DSPE‐mPEG‐DBCO to afford CuSACO with good aqueous dispersibility, blood stability, and bioorthogonal (SPAAC) reactivity. Leveraging the strong optical absorption of graphene and the catalytic activity of Cu single sites, this platform delivers robust photoacoustic signals while simultaneously generating therapeutic ROS [43]. Tostado–Blázquez et al. reported a single‐atom nanozyme in which Cu single atoms are inserted into defect sites of graphene using an energy stimulus (Figure 9c). First, a metal slurry composed of a Cu precursor, a binder, and a carbon source was blade‐coated onto a polyimide substrate and pre‐dried at 80°C to form a thin film. Next, CO_2_ laser scribing directly patterned the film; localized high‐temperature reduction and carbonization converted the organics in the slurry into porous, conductive carbon, while Cu single atoms (Cu‐N_x_/Cu‐C_x_) were immobilized at defect/heteroatom sites to form Cu single‐atom nanozyme tracks. Finally, Ag was sputtered onto the ends of the patterns to create current collectors, and nonactive regions were covered with an insulating layer to complete the working electrode. The resulting Cu SANs electrodes exhibited low detection limits and high sensitivity for analytes such as glucose, dopamine, ascorbic acid, and uric acid [136].

##### 4.2.2.2 Strong Thermal Shock

The strong thermal shock (flash heating or thermal shock) method aims to obtain highly stable SACs by utilizing extremely fast heating and cooling rates to forcibly disperse metal nanoparticles into atomic units and subsequently suppress their re‐aggregation. This approach manufactures SACs via a kinetic route, circumventing thermodynamic equilibrium [137, 138, 139].

##### 4.2.2.3 High‐Energy Ball Milling (HEBM)

High‐energy ball milling (HEBM) utilizes powerful mechanochemical energy to synthesize SACs. This method decomposes and disperses bulk metals or NPs into atomic units, enabling the large‐scale production of SACs [140, 141]. HEBM applies powerful mechanical energy by causing high‐speed collisions, friction, and compression between the balls and the powder sample inside a milling vial. The core principles for SACs synthesis using this method are the forced mechanical dispersion and subsequent anchoring of the metal atoms [142, 143, 144, 145, 146].

## 5 Bioapplication of Stmuli‐Activable SANs

**FIGURE 10:**
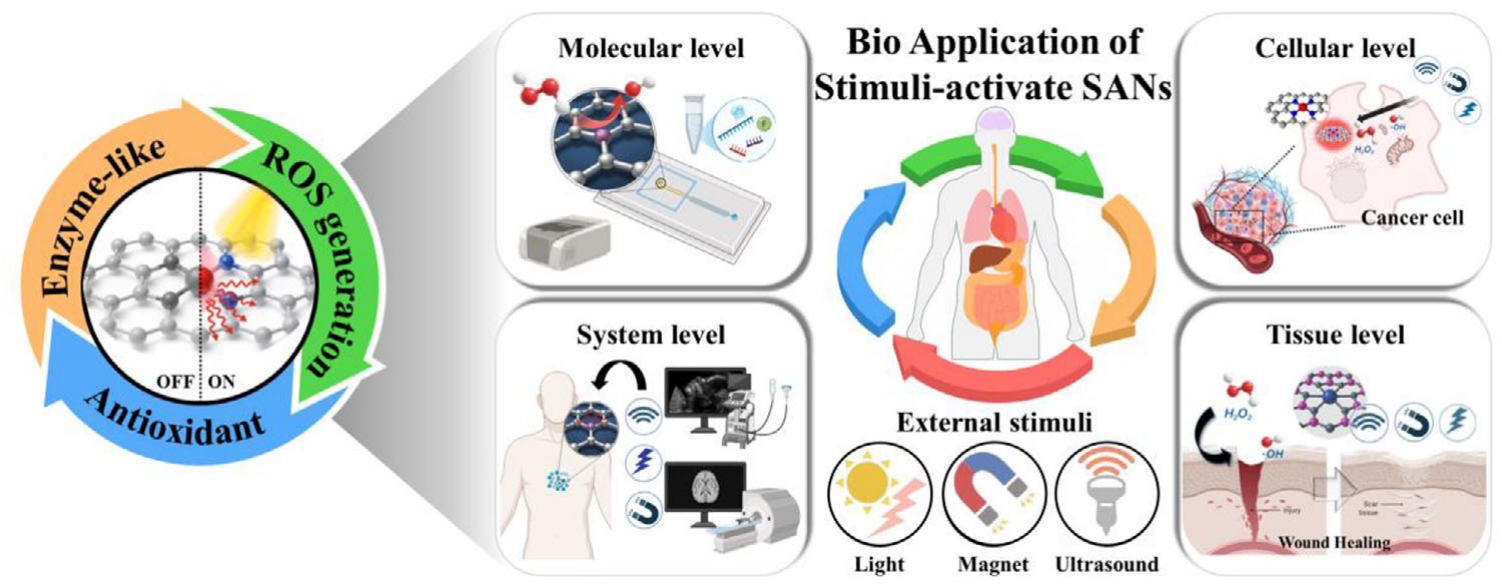
Multiscale illustration of stimuli‐responsive SANs for bio applications. External stimuli regulate catalytic behavior from the molecular to the system level, enabling ROS generation and antioxidants for therapy.

SANs that mimic diverse enzyme‐like activity, such as POD‐like, OXD‐like, SOD‐like, CAT‐like, and GPx‐like, have been widely applied in vitro and in vivo. The key design principle is to tailor their behavior to the disease microenvironment and therapeutic goal, making the direction of ROS amplification (pro‐oxidant) or elimination (antioxidant) explicit. For example, SOD‐like SANs catalyze the dismutation of superoxide radicals (•O_2_^−^), converting them to hydrogen peroxide and molecular oxygen. after this, CAT‐ and GPx‐like SANs remove H_2_O_2_ by converting it to H_2_O and O_2_, thereby alleviating oxidative stress [147]. Conversely, for antimicrobial and antitumor purposes, OXD and POD‐like SANs drive the O_2_ to •OH via the H_2_O_2_ pathway, while CAT‐like O_2_ generation in hypoxic tumor microenvironments replenishes oxygen to sustain and amplify radical production [148]. Integrating SANs with external stimuli maximizes catalytic efficiency, selectivity, and control. For example, color changes arising from SANs‐generated ROS reacting with reporters such as 3,3′,5,5′‐Tetramethylbenzidine (TMB) or 2,2'‐azino‐bis(3‐ethylbenzothiazoline‐6‐sulfonic acid) (ABTS) can be read with high sensitivity to detect various biomolecules. In the cellular microenvironment, external stimuli finely tune ROS amplification and alleviate interfering factors to induce targeted cell death. At the tissue level, excessive ROS can be reduced to relieve inflammation. The opposite *Escherichia coli* (*E. coli)* and other pathogens can be removed by ROS and photothermal effects to reconfigure the healing microenvironment and promote regeneration. Finally, in vivo, remote control (light, ultrasound, magnetic fields) enables site‐specific activation of SANs, and real‐time imaging enables simultaneous diagnosis and therapy (theranostics) (Figure 10a).

### 5.1 Biosensing at the Molecular Level

High‐specificity, high‐sensitivity biosensing is indispensable for disease diagnostics and for analysing diverse molecular species encountered in daily life [149]. Stimuli‐activable SANs offer a compelling solution because their atomically defined reaction sites endow excellent substrate selectivity, while external‐energy activation boosts catalytic turnover, together delivering both enhanced sensitivity and selectivity for accurate and rapid sensing. Recently, leveraging these properties, stimuli‐activable SANs have enabled the development of various sensing methods that are being widely used across diverse biosensing fields.

**FIGURE 11:**
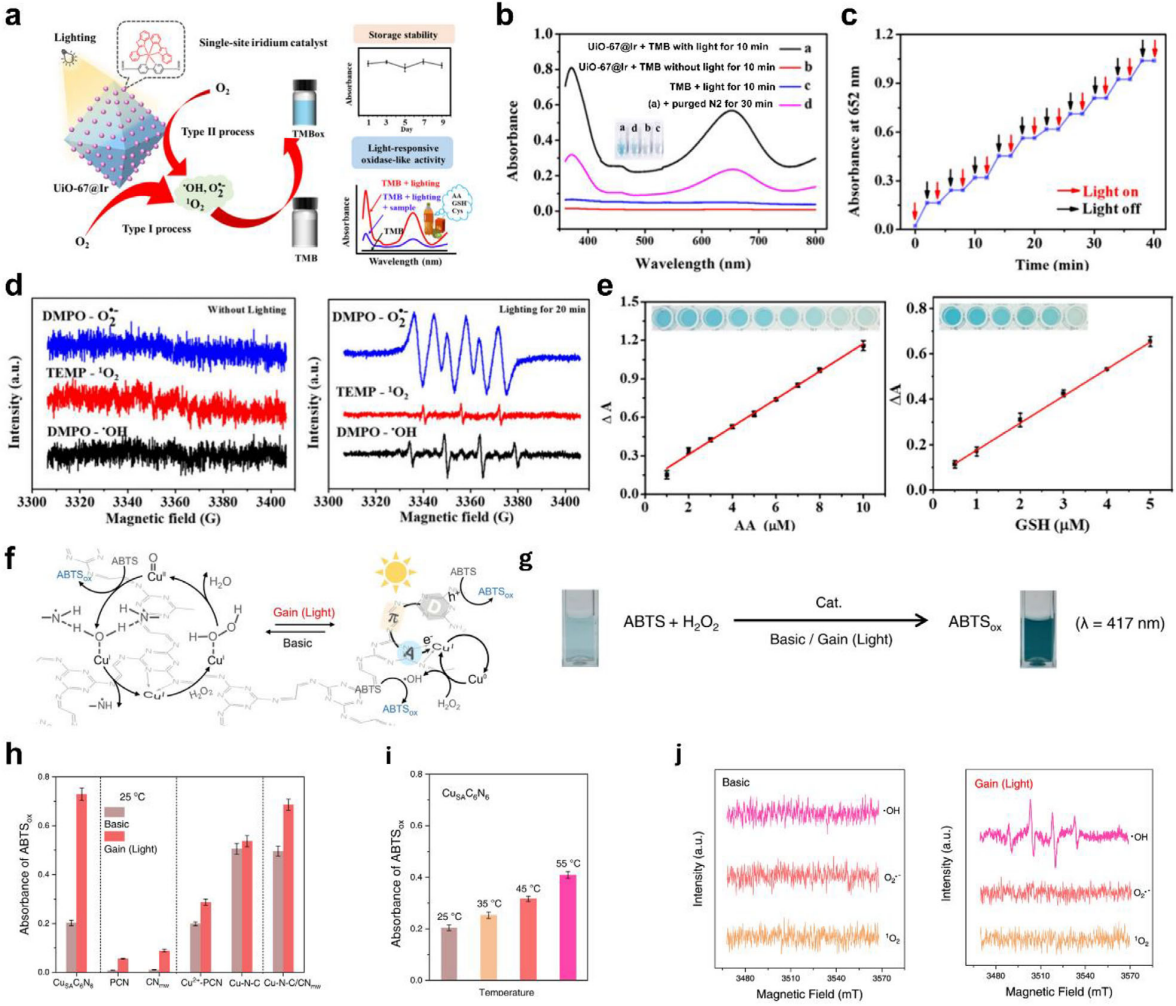
Biosensing. (a) Schematic illustration of light‐responsive oxidase‐like activity for UiO‐67@Ir via type I and type II ROS generation pathways. (b) UV–vis) absorption spectra of four samples. The inset corresponds to pictures of four samples. (c) Staircase‐like behavior of UiO‐67@Ir without and with light, respectively. (d) EPR spectra under different conditions. UiO‐67@Ir with TEMP or DMPO in dark and under light irradiation for 20 min. (e) The plot of the change of absorbance (ΔA) at 652 nm vs AA and GSH concentration. The inset indicates a photograph of the solution at different concentrations of the target. Reproduced with permission [150]. Copyright 2025, American Chemical Society. (f) Proposed mechanism for dual peroxidase‐like and photocatalytic pathways mimicking the basic activity and gain effect using CuSAC_6_N_6_. (g) Equation and photographs of standard ABTS catalytic oxidation using different Catalysts. (h) Absorbance of ABTSox catalyzed by CuSAC_6_N_6_, PCN, CN_mw_, Cu^2+^‐PCN, Cu‐N‐C, and Cu‐N‐C/CN_mw_ without (basic) and with (gain) light irradiation. (i) Absorbance of ABTSox catalyzed by CuSAC_6_N_6_ at different temperatures. ABTS: 2,2’‐azino‐bis (3‐ethylbenzothiazoline‐6‐sulfonic acid). Error bars represent the standard error derived from three independent measurements. (j) EPR spectra of the spin adduct of •OH, •O_2_^−^, and ^1^O_2_ generated during the activation of H_2_O_2_ by CuSAC_6_N_6_ in 0.2 m HAc‐NaAc (pH 5.0) under the basic and gain reactions. Reproduced with permission [151]. Copyright 2022, Springer Nature.

Zhou et al. reported a MOF‐based Ir SANs that exhibits exceptional light‐activable oxidase mimicking activity (Figure 11a–c). Due to abundant active sites dispersed on the light‐responsive supporting materials, irradiation in the presence of O_2_ drives electron transfer to oxygen to generate •O_2_^−^ and •OH, or proceeds via direct energy transfer to produce singlet oxygen (^1^O_2_). These ROS showed efficient oxidation of TMB for a distinct colorimetric signal. Consistent with this mechanism, UiO‐67@Ir produced markedly stronger ROS signals under illumination than in the dark and displayed clear oxidase‐like activity and simultaneously generated •O_2_^−^, •OH, ^1^O_2_ (Figure 11d). The ROS‐mediated TMB oxidation enabled sensitive quantification of ascorbic acid, glutathione, and cysteine, achieving limits of detection of 0.6, 0.5, and 0.3 µm, respectively (Figure 11e). Furthermore, we demonstrate promising sensing capabilities for quantifying the overall antioxidant capacity in fruit and beverage samples [150].

Recently, SANS‐based sensors have used external stimuli to activate multipath way catalysis, boost efficiency, and broaden biosensing applications. Hong et al. reported Cu single‐atom sites immobilized on a graphitic C_6_N_6_ supporting material (Cu_SA_C_6_N_6_) that, under light stimulation, preserve the native catalytic pathway while simultaneously opening an additional ROS‐generation route [151]. In the basic mode(dark), the Cu active sites of Cu_SA_C_6_N_6_ engage in a Cu^2+^/Cu^+^ redox cycle with H_2_O_2_, exhibiting POD‐like activity that generates ROS. During this process, ABTS is oxidized to ABTS_OX_, producing a distinct color change. Upon illumination (gain mode), the Cu_SA_C_6_N_6_ supporting material harvests photons and separates electron‐hole pairs. A fraction of photogenerated electrons is transferred to the Cu centers to accelerate the Cu^2+^/Cu^+^ redox cycle, while photogenerated holes catalyze H_2_O_2_ decomposition, thereby opening an additional ROS pathway such as •OH (Figure 11f). Comparative evaluation of various Cu‐based catalysts revealed that, while Cu^2+^‐PCN and Cu‐N‐C exhibited measurable photoactivity, their gain effects were markedly lower than that of Cu_SA_C_6_N_6_ (Figure 11h). In addition, temperature‐dependent studies showed that catalytic activity increased progressively with reaction temperature, reaching a maximum at 50°C, thereby indicating a potential synergy between thermal and photocatalytic effects (Figure 11i). Subsequently, to verify whether the light indeed opens the gain pathway, we performed electron paramagnetic resonance (EPR) analysis. Comparing the basic mode and the gain mode, we assessed the generation of hydroxyl radicals (•OH), •O_2_^−^, and singlet oxygen (^1^O_2_). In the basic mode, signals for all three species were negligible, whereas in the gain mode, a pronounced •OH signature emerged, clearly evidencing the formation of the light‐initiated general pathway and the associated gain effect (Figure 11j). Leveraging the gain effect, we implemented a glucose sensor. Glucose is oxidized by GOx to generate H_2_O_2_, which is then activated by the Cu_SA_C_6_N_6_ catalyst to oxidize ABTS to ABTS_OX_, producing a colorimetric readout for glucose detection. Upon illumination, the gain effect opens an additional reaction pathway (k_g_), so that, at the same analyte concentration, the sensing sensitivity increases with light intensity. Consequently, while the original POD‐like pathway is preserved, light irradiation opens an additional ROS‐generating route, resulting in a 3.6‐fold increase in catalytic activity relative to the basic mode.

Collectively, these studies highlight that biosensing by stimuli‐activable SANs can improve sensitivity, selectivity, response speed, and signal tunability, while also offering opportunities for theranostics integration. However, the need for additional stimulation devices and the possibility of stimulus‐induced signal interference may increase system complexity, compromise reproducibility under practical conditions, and raise concerns regarding biocompatibility. Therefore, careful optimization of stimulus conditions and system design is essential for reliable and translational biosensing applications.

### 5.2 Cancer Therapy at the Cellular Level

SANs therapy leverages the distinctive conditions of the tumor microenvironment (TME), which include mild acidity, hypoxia, and elevated levels of H_2_O_2_, glucose, and glutathione (GSH) to selectively generate cytotoxic species (e.g., ROS) within tumors, thus minimizing damage to normal tissues, alleviating side effects of conventional therapies, and enabling noninvasive antitumor treatment. In particular, ROS generation in the TME is a central mechanism for inducing tumor cell death, wherein enzyme‐like activities resembling POD, CAT, and OXD produce diverse ROS such as •OH, ^1^O_2_, and •O_2_^−^. However, hypoxia and the low H_2_O_2_ concentration (50–100 µm) constrain ROS generation in the tumor, leaving inherent limitations in selectively killing tumor cells. Consequently, strategies that harness external stimuli such as light, magnetic fields, and ultrasound to efficiently drive catalytic reactions within the TME and selectively kill tumor cells are gaining increasing attention.

**FIGURE 12:**
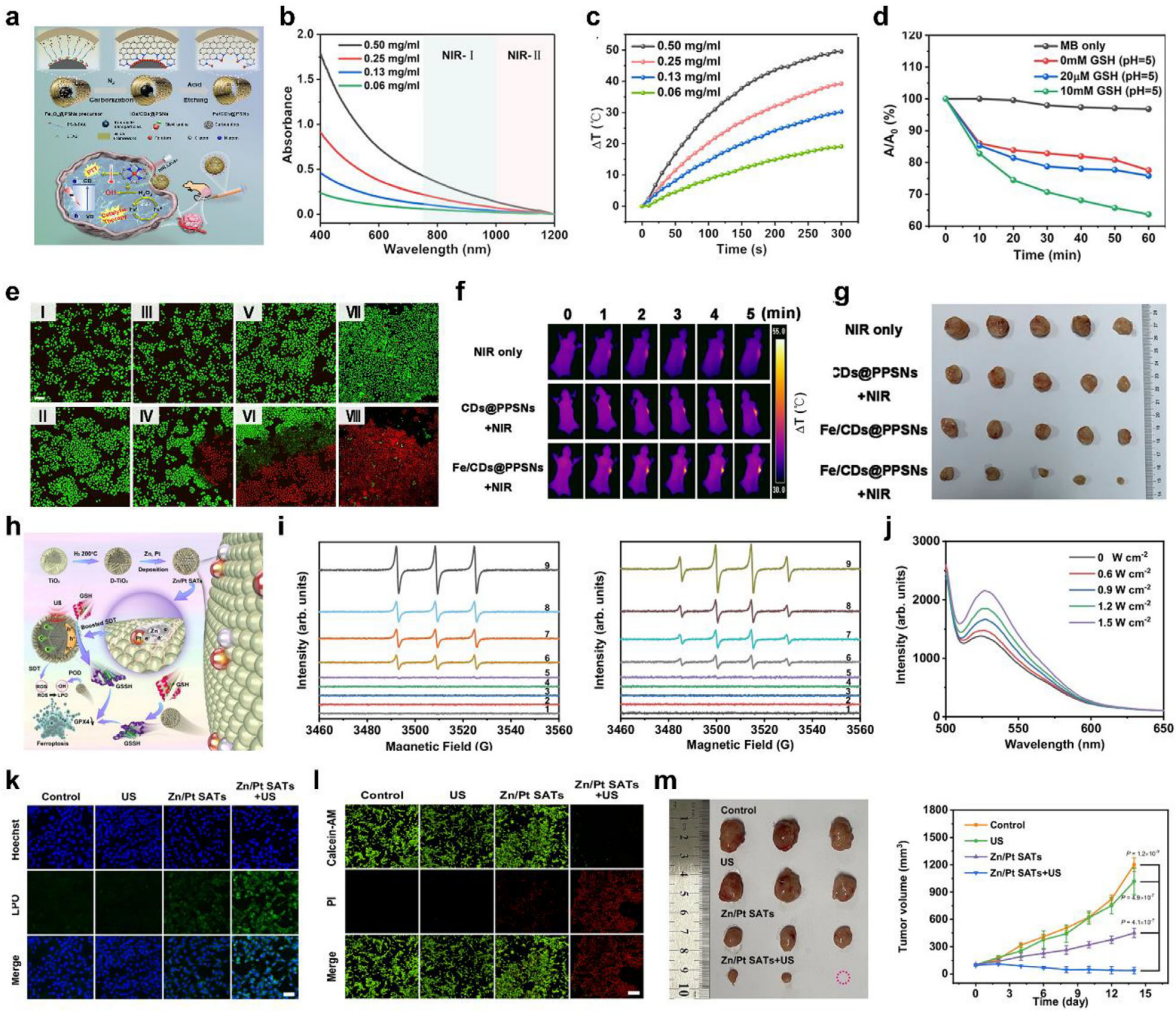
Cancer therapy. (a) Synthetic procedure and therapeutic effects of combined photothermal and catalytic tumor therapy. (b) UV–vis–NIR spectra of Fe/CDs@PPSNs at different concentrations. (c) Photothermal heating curves for Fe/CDs@PPSNs dispersions at different dispersion concentrations (808 nm, 2 W/cm^2^). (d) MB degradation in PBS (10 mm) at various GSH concentrations. (e) Staining of living/dead SMMC‐7721 cells by different treatments: (I) control group, (II) laser irradiation only, (III) CDs@PPSNs, (IV) CDs@PPSNs + NIR, (V) Fe/CDs@PPSNs and (VI) Fe/CDs@PPSNs + NIR, (VII) Fe/CDs@PPSNs + 100 µm H_2_O_2_, (VIII) Fe/CDs@PPSNs+100 µm H_2_O_2_ + NIR, all groups were stained with calcein AM and PI, where green fluorescence from calcein AM and red fluorescence from PI indicate living and dead cells, respectively (scale bar: 50 µm). (f) In vivo infrared thermal imaging images of SMMC‐7721 tumor‐bearing mice treated with PBS, CDs@PPSNs, and Fe/CDs@PPSNs upon 808 nm laser irradiation. (g) Digital photographs of dissected tumors from different groups. Reproduced with permission [88]. Copyright 2022, Springer Nature. (h) Schematic illustration of Zn/Pt SATs sonosensitizer for cancer treatment. (i) Comparison of ^1^O_2_ and •OH generation quantified by ESR spectra of TEMP/^1^O_2_ and DMPO/•OH for different groups of (1) D‐TiO2, (2) Zn SATs, (3) Pt SATs, (4) Zn/Pt SATs, (5) US, (6) D‐TiO_2_ + US, (7) Zn SATs + US, (8) Pt SATs + US, and (9) Zn/Pt SATs + US. (j) Power density‐dependent fluorescence intensity changes of SOSG probe in the presence of Zn/Pt SATs under ultrasound irradiation. (k) Confocal images of LPO detection of 4T1 cells (scale bar: 50 µm). (l) Confocal images of 4T1 cells stained with Calcein‐AM and PI after different treatments. (scale bar: 200 µm). (m) Representative photographs of excised tumors and Tumor growth curves of mice after various treatments (Mean ± S.D., *n* = 5 mice), a two‐tailed *t*‐test was used to calculate P values [152]. Reproduced with permission. Copyright 2024, Springer Nature.

Qin et al. reported an NIR‐responsive SANs designed to exploit tumor‐microenvironment features for enhanced anticancer efficacy. The resulting Fe/CDs@PPSNs comprise a stable architecture in which Fe single atoms are uniformly dispersed within CDs embedded in a porous carbon network formed after silica template removal (Figure 12a). The UV–vis spectrum shows a pronounced absorption band near 400 nm and sustained absorption extending into both NIR‐I (650–950 nm) and NIR‐II (1000–1350 nm) regions. Under NIR laser irradiation, the material exhibits concentration‐dependent photothermal heating, reaching 50°C at 0.5 mg/mL, indicative of efficient photothermal conversion (Figure 12b,c). To determine whether Fe/CDs@PPSNs operate effectively under TME‐like conditions, we conducted pH‐ and GSH‐dependent methylene blue (MB) degradation assays. We first observed that lower pH accelerated the decrease in MB absorbance at λ = 664 nm. Given that GSH in tumor cells can scavenge excess ROS and thereby attenuate nanozyme therapy, we next evaluated MB degradation in the presence of GSH. Notably, Fe/CDs@PPSNs utilized GSH as a reducing agent to convert Fe^3+^ to Fe^2+^, thereby promoting the Fenton‐like reaction; consequently, higher GSH concentrations led to faster and more efficient MB degradation (Figure 12d). This finding suggests that, in the mildly acidic tumor microenvironment, depleting GSH can be leveraged to accelerate catalytic activity and ROS generation. In in vitro experiments with SMMC‐7721 tumor cells, the control (I) exhibited predominantly green fluorescence indicative of viability, whereas the Fe/CDs@PPSNs + H_2_O_2_ + NIR combined treatment (VIII) showed dominant red fluorescence, confirming maximized tumor cell killing. Finally, in a mouse in vivo model, the Fe/CDs@PPSNs + NIR treatment group exhibited superior photothermal conversion efficiency and significant tumor growth inhibition compared with the control groups (Figure 12f,g) [88].

Recently, another external‐stimulus strategy was reported. A Zn/Pt dual‐site SANs that demonstrated synergistic antitumor efficacy. In this nanozyme, Zn and Pt single atoms promote oxygen activation to generate ROS, while Pt single atoms further modulate electron density to accelerate redox reactions and maximize reactivity. Under ultrasound irradiation, the two active sites operate simultaneously, yielding a superimposition‐augmented increase in ROS output compared with a conventional SANs (Figure 12h). In ROS verification using EPR and fluorescent probes, the Zn/Pt dual‐site SAC exhibited markedly stronger ^1^O_2_ and •OH signals under ultrasound irradiation (Figure 12i). Moreover, ROS production increased progressively with time, ultrasound intensity, and co‐substrate (H_2_O_2_) concentration (Figure 12j). These results indicate that the dual active sites induce ROS via distinct pathways yet act complementarily under ultrasound, experimentally validating the unique superimposition‐augmented ROS generation effect. In vitro, the Zn/Pt dual‐site catalyst, under ultrasound stimulation, reduced cancer cell viability in a concentration‐dependent manner, and lipid peroxidation together with ROS generation emerged as the principal drivers of cytotoxicity. The accumulation of ROS and lipid peroxides led to plasma membrane damage, while expression of the ferroptosis suppressor GPX4 was markedly downregulated, clearly substantiating ferroptosis as the dominant cell‐death mechanism (Figure 12k,l). Furthermore, in an in vivo tumor model, the Zn/Pt SANs + ultrasound treatment group achieved the most effective suppression of tumor volume and mass without changes in body weight (Figure 12m) [152]. As another strategy, Fe‐Ag modified quantum dots (QDs)‐based SANs (FAQD) were reported as a representative switchable nanozyme that enables precise catalytic control under ultrasound stimulation. In this construct, Fe mediates Fenton‐like reactions to generate •OH, while Ag facilitates electron transfer, accelerating the Fe^3+^/Fe^2+^ redox cycle. In the quiescent state (no external stimulus), catalytic activity is low, but upon ultrasound, ROS production is triggered immediately, and this ultrasound‐induced oxidation can be toggled on/off. Consequently, excess oxidative stress accumulates in tumor cells, inducing cell death, whereas unnecessary activation is avoided in normal tissues, conferring high therapeutic selectivity. Moreover, Ag doping narrows the bandgap and introduces dopant states, promoting electron‐hole separation and enhancing ^1^O_2_ generation under ultrasound, as verified by EPR. Simultaneously, atomically dispersed Fe undergoes ultrasound‐activated electron transfer and is reduced from Fe^3+^ to Fe^2+^, after which H_2_O_2_ is converted to •OH via the Fenton‐like pathway. Practically, FAQD showed a pronounced increase in dissolved O_2_ attributable to H_2_O_2_ decomposition, together with a time‐dependent rise in EPR signals relative to controls. Under ultrasound phenanthroline assays, which selectively complex with Fe^2+^ to yield a characteristic 510 nm absorbance, confirmed Fe^3+^/Fe^2+^ interconversion, and RNO bleaching verified ROS generation. Furthermore, at both the cellular and in vivo levels, robust tumor suppression was observed exclusively in the FAQD + US group [153].

In conclusion, these studies demonstrate that stimuli‐activable SANs can enhance ROS generation with improved spatiotemporal controllability, tumor selectivity, and synergistic therapeutic efficacy in the tumor microenvironment. However, their performance may still be limited by heterogeneous TME conditions, insufficient stimulus penetration, and the risk of off‐target oxidative damage. Therefore, careful optimization of stimulus parameters and catalyst design is essential to maximize antitumor efficacy while minimizing adverse effects on normal tissues.

### 5.3 Tissue Regeneration at the Tissue Level

In clinical practice, infectious diseases and tissue injury frequently co‐occur, and antibiotic‐resistant bacteria in particular can severely undermine the efficacy of conventional therapies. Bacterial proliferation and the ensuing chronic inflammation impede healing and regeneration, further complicating treatment [154]. To address these limitations, externally stimulated single‐atom catalytic systems are gaining attention. By harnessing light, ultrasound, magnetic fields, and related stimuli to selectively generate ROS [155], they can achieve efficient bactericidal activity while attenuating inflammation and promoting tissue regeneration, thereby providing a promising alternative to existing approaches.

**FIGURE 13:**
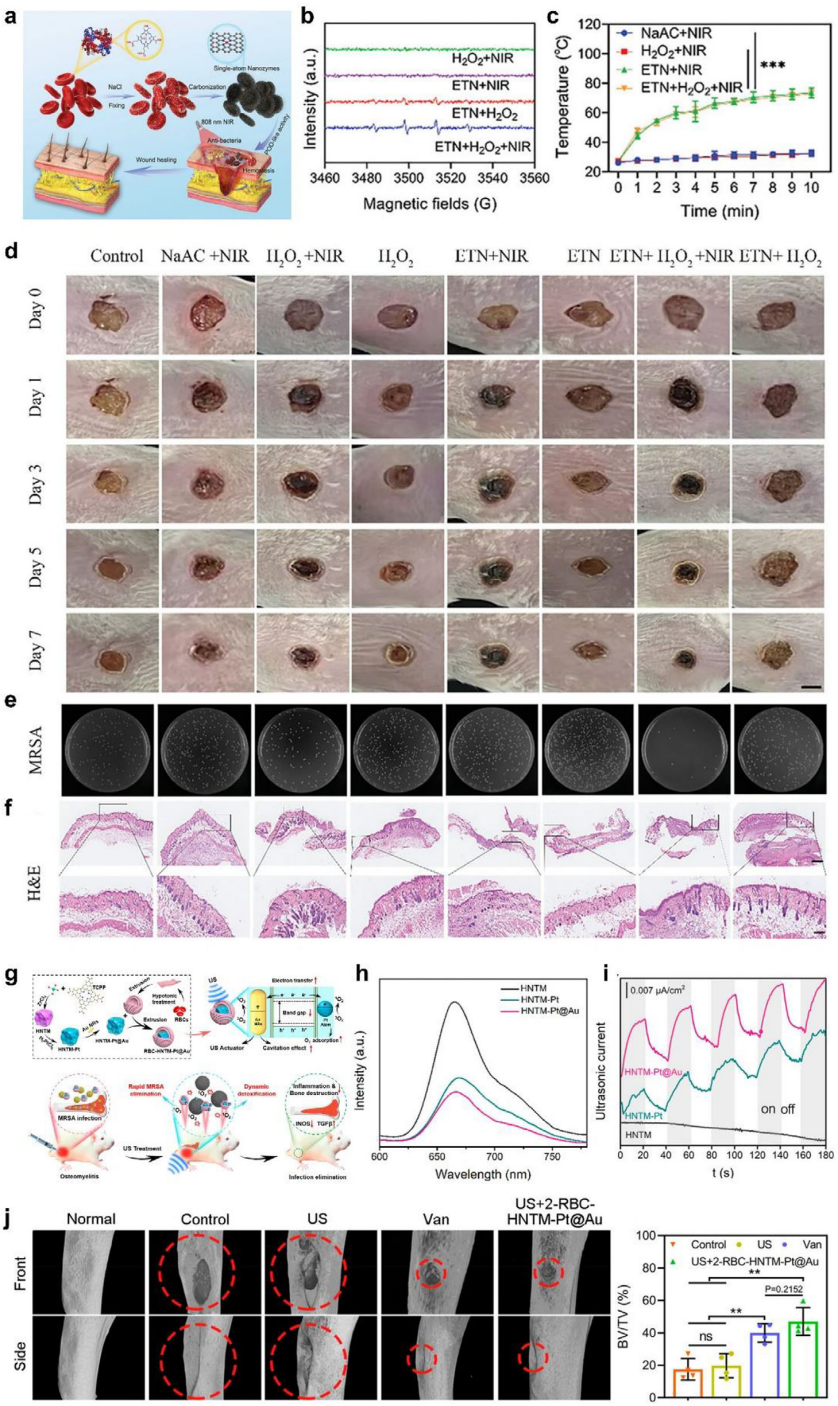
Tissue regeneration. (a) The schematic of using erythrocytes to make single‐atom nanozymes with multifunction properties for wound healing. (b) ESR spectra of DMPO/NaAC solution upon addition of H_2_O_2_ + NIR (2.0 W/cm^2^, 6 min), ETN + NIR, ETN + H_2_O_2_, or ETN + H_2_O_2_ + NIR. (c) Temperature‐elevating curves of different treatments. (d) Time‐dependent photographs of wounds in mice under different treatments. Scale bars = 4 mm. (e) Agar plate images of antibacterial effects under different treatments for 7 days. (f) H&E staining of wound tissues in different groups. ^*^ *p* < 0.05. Scale bars = 10 µm (up) and 200 µm (down). Reproduced with permission [156]. Copyright 2023, Wiley‐VCH GmbH. (g) Synthesis of the RBC‐HNTM‐Pt@Au, sonocatalytic mechanism, and the treatment of osteomyelitis through efficient SDT. (h) Photoluminescence spectra of HNTM, HNTM‐Pt, and HNTM‐Pt@Au. (i) Photoluminescence spectra of HNTM, HNTM‐Pt, and HNTM‐Pt@Au. (j) Micro‐CT images of tibial defects after 4 weeks of treatment. (k) BV/TV values (*n* = 4 independent experiments per group, ^**^ *p* < 0.01). Reproduced with permission [87]. Copyright 2021, American Chemical Society.

Wang et al. synthesized an erythrocyte‐templated nanozyme (ETN) bearing Fe single‐atom active sites and demonstrated NIR‐assisted healing of infected wounds. Fe single atoms are anchored within a porous N‐doped carbon framework produced by a NaCl confined carbonization process leveraging its NIR absorption and POD/SOD‐like activities, ETN under 808 nm irradiation converts photothermal energy and H_2_O_2_ into •OH, thereby amplifying bactericidal efficacy (Figure 13a,b). The nanozyme's radical‐generating capacity and photothermal performance were verified by EPR and temperature profile measurements: in the ETN + H_2_O_2_ + NIR condition, EPR signals were maximized, confirming enhanced •OH production, and temperature profiles showed rapid heating to 60°C–70°C within minutes (Figure 13c). Consistently, DCFH‐DA assays revealed a surge in ROS for ETN + H_2_O_2_ + NIR, accompanied by GSH depletion. This catalytic reaction (ROS amplification + photothermal damage + GSH consumption) translated in a Methicillin‐resistant *Staphylococcus aureus* (MRSA) infected wound model to the fastest wound size reduction, a marked drop in colony forming unit at the infection site, and H&E histology indicating reduced inflammatory cell infiltration with aligned collagen remodeling and tissue restoration (Figure 13d–f) [156].

Yu et al. reported a treatment for deep‐tissue infections that combines the strengths of ultrasound with single‐atom catalysis for. The team synthesized a catalyst with Pt single atoms immobilized on a MOF supporting material and coupled it with Au nanorods to construct HNTM‐Pt@Au. While conventional ultrasound‐based therapies offer good tissue penetration, they are often limited by insufficient O_2_ supply and low ROS yields. By contrast, the rationally engineered HNTM‐Pt@Au promotes O_2_ adsorption/activation at Pt active sites under ultrasound, and the Au nanorods enhance ultrasound energy absorption and charge transfer, thereby efficiently driving a continuous cascade of electron capture → O_2_ adsorption → ROS generation to achieve potent antibacterial activity and tissue regeneration (Figure 13g). Following Pt single‐atom doping, the HOMO‐LUMO gap was tuned from 1.90 to 1.80 eV, modifying the electronic structure and facilitating charge separation and transport. Consistently, PL quenching evidenced suppressed electron‐hole recombination (Figure 13h,i). On this basis, HNTM‐Pt@Au exhibited the strongest ultrasound‐responsive current, and DPBF assays confirmed the highest ^1^O_2_ production among comparators. Under ultrasound, HNTM‐Pt@Au markedly reduced bacterial colony counts relative to controls, and Live/Dead staining showed a pronounced increase in red fluorescence indicative of killed bacteria. Micro‐CT analysis showed marked structural restoration of infection‐damaged bone in the US + 2‐RBC‐HNTM‐Pt@Au group. In contrast, the control and US‐only groups exhibited extensive bone loss and vancomycin treatment achieved only partial improvement. By comparison, the US + 2‐RBC‐HNTM‐Pt@Au group recovered toward normal bone architecture, with a significant increase in BV/TV, demonstrating the best bone regenerative outcome. These results indicate that the synergy between single‐atom catalysis and ultrasound drives not only antibacterial effects but also bone remodeling and tissue repair (Figure 13j,k) [87].

Collectively, stimuli‐activable SANs provide a promising strategy for tissue regeneration by combining efficient antibacterial activity with inflammation attenuation and regenerative support. Nevertheless, excessive ROS generation or uneven stimulus delivery may damage surrounding healthy tissues and compromise reproducibility in practical applications. Thus, achieving a precise balance between antimicrobial efficacy and tissue compatibility will be critical for successful clinical translation.

### 5.4 Theranostics at the System Level

The persistent burden of postoperative complications and the ever‐increasing annual volume of surgeries underscore the limitations of conventional invasive approaches [157]. Consequently, there is a pressing need to develop noninvasive in vivo manipulation and therapy. One avenue has explored external stimulation therapy (photothermal, acoustic modulation), yet challenges remain in efficacy and precise control [158]. To address these gaps, SANs‐based, noninvasive in vivo therapeutic strategies driven by external stimulation (ultrasound, magnetic fields, and light) are gaining attention. This approach reengineers the electronic structure, spin state, and reaction pathway at single active sites and integrates defect, coordination, and supporting material engineering, thereby enabling fine control of ROS and photothermal outputs under low intensity stimulation and high efficiency catalytic reactions. Coupled with NIR‐II, MRI, CT, ultrasound, and photoacoustic imaging, it can simultaneously achieve spatiotemporal precision and reduced systemic toxicity [159]. As a result, by establishing a theranostics (simultaneous diagnosis‐therapy) system, this approach demonstrates the feasibility of noninvasive treatment with concurrent diagnosis.

**FIGURE 14:**
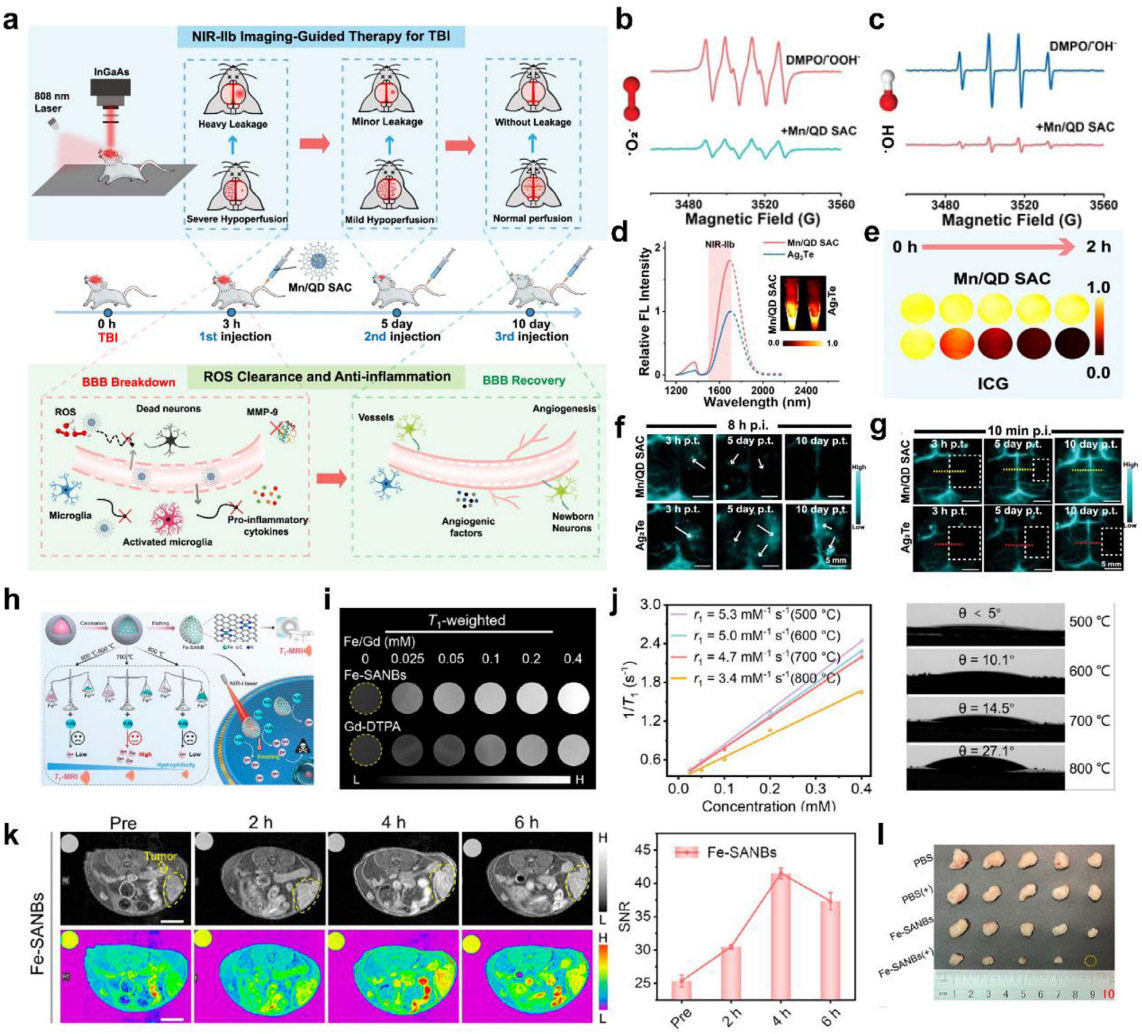
Theranostics. (a) The near‐infrared‐IIb emitting Mn single‐atom catalyst enables dynamically monitoring the blood–brain barrier (BBB) status in a noninvasive way and repressing the ROS‐mediated BBB breakdown. (b) ESR spectra of •O_2_^−^ with Mn/QD SAC using DMPO as the spin trap agent. (c) ESR spectra of •OH with Mn/QD SAC using DMPO as the spin trap agent. (d) Fluorescence emission spectra of Mn/QD SAC and Ag_2_Te QDs. The inset images were the NIR‐IIb images of Mn/QD SAC and Ag_2_Te QDs, respectively. (e) Fluorescence images of Mn/QD SAC and ICG under the 808 nm laser irradiation, which were taken at different time points (0, 30, 60, 90, and 120 min). (f) NIR‐IIb imaging of the brain of TBI mice performed within 8 h post‐injection with Mn/QD SAC or Ag_2_Te at 3 h, fifth, or 10th day post‐TBI. The white arrow points to the location of the leakage. (g) NIR‐IIb imaging of the brain of TBI mice performed within 10 min post‐injection with Mn/QD SAC or Ag_2_Te at 3 h, 5‐th, or 10th day post TBI. The white dotted line frames the ischemic areas of the brain [160]. Reproduced with permission. Copyright 2023, Springer Nature. (h) Illustration of Single‐Atom Iron Nanobowls (Fe‐SANBs) with Tunable Fe(II)/Fe(III) Ratios for MRI‐Guided Cancer Therapy with NIR Light‐Enhanced CDT Effects. (i) T_1_‐weighted phantom MRI with varying concentrations of Fe‐SANBs and Gd‐DTPA at 3 T. (j) The r_1_ at 7 T of samples treated at different temperatures, and Schematic snapshots showing the contact angles of water droplets for samples treated at different temperatures. (k) In vivo ^1^H MRI images of 4T1‐tumor‐bearing mice at different time points after intravenous administration of Fe‐SANBs (10 µmol/kg Fe of body weight) and Quantification of Fe‐SANBs MRI signal‐to‐noise ratios in tumors of mice. (l) Tumor excision photograph on the 14th day of treatment. Reproduced with permission [83]. Copyright 2024, American Chemical Society.

Huang et al. synthesized Mn single‐atom doped Ag_2_Te quantum dots (Mn/QD SANs) with NIR‐IIb emission and harnessed them to build an in vivo theranostics platform that integrates diagnosis and therapy. At single active sites, Mn^2+^/Mn^3+^ redox cycling mediates CAT/SOD‐like pathways that sequentially scavenge H_2_O_2_, •O_2_^−^, and •OH (Figure 14a). The CAT‐like activity was validated by H_2_O_2_ decomposition kinetics, showing concentration‐dependent increases in the initial slope of the dissolved‐oxygen (ΔDO)‐time curves, and quantitatively confirmed by reduced •OOH/•OH signals in DMPO‐EPR (Figure 14b,c). Optically, the Mn/QD SANs deliver stronger emission and superior vascular contrast in the NIR‐IIb range (1500–1700 nm) compared with Ag_2_Te, and exhibit markedly better signal retention than ICG under 808 nm irradiation, enabling extended live imaging (Figure 14d,e). Based on these properties, the authors evaluated a traumatic brain injury model at 3 h, 5 days, and 10 days post‐injury. 10 min post‐injection, imaging readouts of reperfusion/ischemia showed a reduced ischemic area, indicating improved early reperfusion at 8 h p.i., decreased background leakage with preserved vessel delineation indicated blood‐brain barrier stabilization (Figure 14f,g). Overall, Mn/QD SANs integrate potent antioxidant nanozyme activity with high‐contrast short‐wave infrared (NIR‐IIb, 1500–1700 nm) emission into a single platform, enabling simultaneous diagnosis and therapy. This theranostics strategy supports precise, noninvasive treatment under low external stimulation [160]. Luo et al. designed hydrophilic nanobowls featuring Fe‐N_4_ single‐atom active sites (Fe‐SANBs) to realize T1‐MRI contrast and CDT within a single platform (Figure 14h). The concave nanobowl architecture facilitates water access to the metal center. Accordingly, under the Solomon‐Bloembergen‐Morgan (SBM) theory, the under‐coordinated Fe‐N_4_ increases the number of water molecules bound (q), while the nanobowl geometry accelerates water exchange (τM), thus shortening the T_1_ relaxation time. Experimentally, FeSANBs exhibited a longitudinal relaxivity (r_1_) of 11.48 m/Ms, approximately 3.1‐fold higher than the clinical agent Gd‐DTPA (3.72 m/Ms). Consistently, T_1_‐weighted phantom MRI showed faster signal enhancement at identical concentrations for Fe‐SANBs (Figure 14i). Pyrolysis optimization identified 700°C as the condition that simultaneously maximized r_1_ and CDT efficacy. Specifically, at 500–600°C, a lower Fe^2+^ and Fe^3+^ ratio afforded strong T_1_‐MRI performance but limited •OH generation, whereas at 800°C, diminished surface hydrophilicity reduced both T_1_‐MRI contrast and •OH yield. Thus, 700°C emerged as the optimal balance for contrast performance and CDT activity (Figure 14j). In addition, the photothermal (PTT) effect of Fe‐SANBs accelerated •OH production within the tumor microenvironment, further enhancing CDT (Fig. e). In vivo T_1_‐MRI in 4T1 tumor‐bearing mice showed progressive tumor signal enhancement after intravenous injection, peaking at 4 h and declining modestly by 6 h (Figure 14k). Signal‐to‐noise ratio (SNR) quantification mirrored this temporal profile, and compared with Gd‐DTPA, Fe‐SANBs exhibited a slower washout (shallower decay slope), yielding a longer duration of effective contrast. Therapeutically, complementary PTT and CDT produced significant tumor growth inhibition relative to controls (Figure 14l). Overall, Fe‐SANBs deliver high‐contrast T_1_‐weighted MRI, excellent biocompatibility, and robust antitumor efficacy, establishing an externally responsive single‐atom nanoplatform that enables high contrast T_1_‐MRI diagnosis and imaging‐guided cancer therapy in one system [83].

Overall, stimuli‐activable SANs theranostics platforms highlight the potential of integrating precise diagnosis and therapy within a single system, enabling spatiotemporally controlled treatment with reduced systemic toxicity. However, challenges remain in platform complexity, signal reliability, in vivo biosafety, and the standardized control of stimulus delivery. Accordingly, further optimization of nanoplatform engineering and treatment protocols will be necessary to realize robust and clinically applicable theranostics systems.

## 6 Summary and Perspective

Stimuli‐activable SANs utilize external stimuli to precisely regulate isolated atomic active sites, thereby enhancing catalytic performance and demonstrating strong potential in biosensing, disease therapy, and imaging. These activation strategies enable switchable control over catalytic activity and remote controllability, while reducing off‐target toxicity and unnecessary catalyst consumption through spatiotemporal confinement. These systems, when combined with microenvironment responsivity and molecular targeting, can further enhance selectivity and therapeutic efficacy in cancer and other diseases. Moreover, multimodal designs that integrate PDT, PTT, and ultrasound strengthen imaging‐therapy synergy for more effective treatment. Despite these advantages, the clinical translation of SANs requires overcoming several fundamental and technical challenges.

First, biocompatibility must be prioritized to fully realize the potential of nanozymes. Some SANs may induce toxic side effects due to unintended release or diffusion, and comprehensive safety data remain limited. Therefore, intrinsic safety should be incorporated into material design, for instance, through eco‐friendly biosynthetic approaches (e.g., plant extracts, algae, yeast) or cell‐membrane coatings to improve biocompatibility. In addition, biodegradability must be considered, as degradation processes may generate toxic byproducts that adversely affect healthy tissues. Comprehensive evaluation of both short‐ and long‐term biocompatibility, along with in vivo degradation pathways and products, is therefore essential.

Second, broader biomimicry of natural metalloenzymes is required to expand the functional scope of SANs. Current systems primarily rely on ROS generation and scavenging, which limits their catalytic diversity. By extending biomimetic strategies from natural metalloenzymes to engineered SANs, a wider range of catalytic functionalities and more precise activity regulation can be achieved. In particular, integrating multiple enzyme‐like functions within a single system can enable cooperative and sequential catalytic processes, thereby enhancing overall catalytic efficiency and enabling more sophisticated reaction regulation.

Third, precise and selective control of catalytic activity in response to external stimuli requires advances in catalyst design and operating mechanisms. SANs can enable remote, site‐specific switching of catalytic activity with high precision to minimize damage to healthy tissues. To ensure the accuracy of such control, diagnostic imaging should be integrated to confirm activation at the intended site and enable real‐time monitoring of therapeutic processes. This integration further enables SANs to develop into remotely controlled theranostics systems combining targeted activation with noninvasive imaging modalities such as NIR fluorescence, photoacoustic imaging, and MRI.

Fourth, integrating multiple external stimuli into SAN systems is essential for enhancing catalytic controllability and performance. While most SAN systems rely on a single external stimulus, integrating multiple stimuli enables functional differentiation, where distinct stimuli can be assigned complementary roles such as catalytic activation and imaging guidance. Such an approach can improve spatial precision, targeting accuracy, and catalytic efficiency, while enabling the integration of diagnostic and therapeutic functions.

Finally, most stimuli‐activable SANs systems are primarily focused on enhancing overall catalytic efficiency rather than selectively controlling specific reaction pathways. Recent studies have demonstrated that introducing chiral structures and spin‐related effects can enhance reaction selectivity under external stimuli, enabling preferential activation of specific catalytic pathways. Therefore, integrating external stimuli with rational structural design to achieve selective catalytic regulation represents an important direction for improving the specificity of SANs systems. This will enable standardized dosing, stimulation protocols, and reproducibility supported by companion diagnostics.

In summary, stimuli‐activable SANs enable on‐demand switching of catalytic activity under external stimuli, providing precise spatiotemporal control and strong potential in biosensing, cancer and infection therapy, and imaging. For successful clinical translation, minimizing off‐target toxicity and enabling real‐time monitoring of therapeutic responses are critical. This requires the development of remotely controlled theranostics systems that combine target‐selective activation and deactivation with noninvasive, imaging‐guided control. Furthermore, a systematic development framework encompassing biocompatibility, degradability, stimulation standardization, targeting strategies, large‐animal validation, and reproducibility will be essential for advancing SANs toward practical biomedical applications.

## Conflicts of Interest

The authors declare no conflicts of interest.

## Data Availability

The data that support the findings of this study are available on request from the corresponding author. The data are not publicly available due to privacy or ethical restrictions.
